# Bispecific killer cell engagers employing species cross-reactive NKG2D binders redirect human and murine lymphocytes to ErbB2/HER2-positive malignancies

**DOI:** 10.3389/fimmu.2024.1457887

**Published:** 2024-08-29

**Authors:** Jordi Pfeifer Serrahima, Katrin Schoenfeld, Ines Kühnel, Julia Harwardt, Arturo Macarrón Palacios, Maren Prüfer, Margareta Kolaric, Pranav Oberoi, Harald Kolmar, Winfried S. Wels

**Affiliations:** ^1^ Georg-Speyer-Haus, Institute for Tumor Biology and Experimental Therapy, Frankfurt, Germany; ^2^ Frankfurt Cancer Institute, Goethe University, Frankfurt, Germany; ^3^ Institute for Organic Chemistry and Biochemistry, Technical University of Darmstadt, Darmstadt, Germany; ^4^ Centre for Synthetic Biology, Technical University of Darmstadt, Darmstadt, Germany; ^5^ German Cancer Consortium (DKTK), Partner Site Frankfurt/Mainz, Frankfurt, Germany

**Keywords:** bispecific killer cell engager, BiKE, NKG2D, ErbB2, HER2, natural killer cells, NK-92, chimeric antigen receptor

## Abstract

NKG2D is an activating receptor expressed by natural killer (NK) cells and other cytotoxic lymphocytes that plays a pivotal role in the elimination of neoplastic cells through recognition of different stress-induced cell surface ligands (NKG2DL). To employ this mechanism for cancer immunotherapy, we generated NKG2D-engaging bispecific antibodies that selectively redirect immune effector cells to cancer cells expressing the tumor-associated antigen ErbB2 (HER2). NKG2D-specific single chain fragment variable (scFv) antibodies cross-reactive toward the human and murine receptors were derived by consecutive immunization of chicken with the human and murine antigens, followed by stringent screening of a yeast surface display immune library. Four distinct species cross-reactive (sc) scFv domains were selected, and reformatted into a bispecific engager format by linking them via an IgG4 Fc domain to a second scFv fragment specific for ErbB2. The resulting molecules (termed scNKAB-ErbB2) were expressed as disulfide-linked homodimers, and demonstrated efficient binding to ErbB2-positive cancer cells as well as NKG2D-expressing primary human and murine lymphocytes, and NK-92 cells engineered with chimeric antigen receptors derived from human and murine NKG2D (termed hNKAR and mNKAR). Two of the scNKAB-ErbB2 molecules were found to compete with the natural NKG2D ligand MICA, while the other two engagers interacted with an epitope outside of the ligand binding site. Nevertheless, all four tested scNKAB-ErbB2 antibodies were similarly effective in redirecting the cytotoxic activity of primary human and murine lymphocytes as well as hNKAR-NK-92 and mNKAR-NK-92 cells to ErbB2-expressing targets, suggesting that further development of these species cross-reactive engager molecules for cancer immunotherapy is warranted.

## 1 Introduction

Bispecific antibodies that crosslink activating receptors on cytotoxic lymphocytes with tumor-associated surface antigens hold enormous potential for targeted cancer immunotherapy. While bispecific T-cell engagers (BiTEs) such as blinatumomab selectively redirect T lymphocytes to cancer cells via binding to CD3 (1), bispecific and trispecific killer cell engagers (BiKEs, TriKEs) which interact with the IgG-binding receptor CD16a (FcγRIIIa), natural cytotoxicity receptors (NCRs), or the C-type lectin-like protein Natural Killer Group 2D (NKG2D) predominantly recruit innate killer cells to the tumor (2–4). With respect to the latter, most progress has been made with bispecific antibodies that activate CD16a-positive natural killer (NK) cells, and several such molecules are currently evaluated in clinical trials for the treatment of lymphoma, advanced solid cancers, multiple myeloma (MM) or acute myeloid leukemia (AML) (5). In a different approach, Gaulthier and colleagues designed TriKEs with further enhanced activating potential by combining an IgG1 Fc region for engagement of CD16a with an antibody fragment specific for NKp46, a natural cytotoxicity receptor almost exclusively expressed on NK cells, and a tumor-targeting domain recognizing antigens like CD123 on AML cells (6). Similar to NCRs, NKG2D represents a promising activating receptor for engager-mediated redirection of cytotoxic lymphocytes to cancer cells, with different NKG2D-targeted approaches being under active development (2, 7).

NKG2D is not only expressed by NK cells, but also NKT cells, CD8^+^ T cells, and subpopulations of CD4^+^ and γδ T cells, and is critically involved in the immunosurveillance of malignancies and pathogens (8, 9). In humans, NKG2D recognizes eight stress-induced cell surface ligands (NKG2DL) that are expressed by almost all cancer types, and include the MHC class I-related molecules MICA and MICB, as well as the six UL16-binding proteins ULBP1 to ULBP6 (10, 11). Nevertheless, low or absent NKG2DL expression by leukemia-initiating cells (12), and removal of NKG2DL from the cell surface by proteolytic shedding can still result in escape of cancer cells from NKG2D-mediated immune surveillance (13–15). Bispecific NKG2D engagers circumvent this dependency on NKG2DL expression, and redirect NKG2D-positive effector lymphocytes to tumor cells irrespective of the presence of natural ligands. For NKG2D binding, such recombinant molecules employ NKG2D-specific single chain fragment variable (scFv) antibodies or nanobodies (16–20), or domains derived from natural NKG2D ligands like ULBP2 or MICA (21–24). These NKG2D-engaging units are linked to a second binding domain targeting surface antigens expressed by hematological malignancies or solid tumors.

Like all engagers of this architecture, bispecific NKG2D antibodies depend on the quality and effectiveness of the cytotoxic lymphocytes they recruit to the tumor. However, in cancer patients endogenous NK cells are often functionally compromised. Therefore, *ex vivo* expanded NK cells from healthy donors are typically employed for adoptive NK cell immunotherapies (25, 26). Also the continuously expanding human NK cell line NK-92 and chimeric antigen receptor (CAR)-engineered derivatives thereof are being developed for clinical applications (27–30). Unlike allogeneic T lymphocytes, unrelated donor NK cells do not induce graft-*versus*-host disease (GvHD), even if applied in an HLA-unmatched setting (25). This makes them ideal candidates for the development of safe and cost-effective off-the-shelf therapeutics. Consequently, to enhance the efficacy of bispecific killer cell engagers that interact with CD16a or NKG2D, strategies have been devised to combine them with adoptive transfer of CD16a-positive donor NK cells (31), or allogeneic NK or T cells engineered with an NKG2D-derived CAR (18, 20).

To expand the armamentarium of NKG2D binders suitable for engineering effective engager molecules, here we immunized chicken with human and murine NKG2D, generated a yeast surface display immune library and selected a panel of four novel avian scFv antibody fragments, which in contrast to current molecules not only activate human NKG2D but also its murine homolog. The species cross-reactive (sc) binding domains were then employed to derive bispecific NKG2D-activating antibodies (termed NKABs) by fusing them via an IgG4 Fc region to a second scFv fragment which targets the tumor-associated antigen ErbB2 (HER2). Utilizing NKG2D-positive primary human and murine lymphocytes as well as established NK-92 cells engineered to express NKG2D-derived CARs (termed NKARs) either based on human or murine NKG2D, we investigated binding of the resulting scNKAB-ErbB2 molecules to human and murine NKG2D receptors, competition with the natural NKG2D ligand MICA and redirection of the effector lymphocytes to ErbB2-expressing cancer cells in comparison to reference NKAB-ErbB2 molecules that interact only with human or murine NKG2D.

## 2 Materials and methods

### 2.1 Cells and culture conditions

MDA-MB-453 and MDA-MB-468 breast carcinoma, and EL-4 T-cell lymphoma cells (all ATCC, Manassas, VA) were cultured in DMEM (Gibco, Thermo Fisher Scientific, Darmstadt, Germany), A20 B-cell lymphoma and K562 erythroleukemia cells (both ATCC), and CEM.NKR and RMA/neo T lymphoblastoid cells (kindly provided by Alexander Steinle, Goethe University, Frankfurt, Germany) in RPMI 1640 medium (Gibco, Thermo Fisher Scientific). All media were supplemented with 10% heat-inactivated FBS (Capricorn Scientific, Ebsdorfergrund, Germany), 2 mM L-glutamine, 100 U/mL penicillin and 100 µg/mL streptomycin (all Gibco), and 50 µM β-mercaptoethanol (Sigma-Aldrich, Merck, Darmstadt, Germany) for A20 cells. Expi293F embryonic kidney cells were cultured in Expi293 expression medium (both Gibco, Thermo Fisher Scientific). NK-92 cells (32) (kindly provided by NantKwest, Inc., Culver City, CA) and genetically engineered derivatives thereof were grown in X-VIVO 10 medium (Lonza, Cologne, Germany) supplemented with 5% heat-inactivated human AB plasma (German Red Cross Blood Donation Service Baden-Württemberg-Hessen, Frankfurt, Germany) and 100 IU/mL IL-2 (Proleukin; Novartis Pharma, Nürnberg, Germany). Peripheral blood mononuclear cells (PBMCs) were isolated from commercially obtained buffy coats of anonymous blood donors (German Red Cross Blood Donation Service Baden-Württemberg-Hessen) by Ficoll-Hypaque density gradient centrifugation, and cultured in X-VIVO 10 medium supplemented with 5% heat-inactivated human AB plasma, 500 IU/mL IL-2 and 50 ng/mL hIL-15 (Peprotech, Hamburg, Germany). Murine NK cells were isolated from splenocytes derived from C57BL/6 mice by MACS separation using the NK Cell Isolation Kit (Miltenyi Biotec, Bergisch Gladbach, Germany). Splenocytes and murine NK cells were kept in RPMI 1640 medium supplemented with 50 µM β-mercaptoethanol and 20 ng/mL mIL-15 (Miltenyi Biotec).

### 2.2 Generation of recombinant NKG2D proteins and immunization of chicken

Constructs for expression of NKG2D-Fc fusion proteins were generated by assembling sequences encoding an immunoglobulin heavy chain signal peptide, the extracellular domain of human NKG2D (hNKG2D; UniProtKB: P26718, amino acid residues 82-216) or murine NKG2D (mNKG2D; UniProtKB: Q2TJJ6, amino acid residues 98-232), and either hinge, CH2 and CH3 domains of human IgG4 in plasmid pcDNA3, or a Strep-Tag II, a 6xHis-Tag, a Tobacco Etch Virus (TEV) cleavage site and hinge, CH2 and CH3 domains of human IgG1 in plasmid pTT5. Expi293F cells were then transiently transfected with the resulting vectors using the ExpiFectamine 293 Transfection Kit according to the manufacturer’s recommendations (Gibco, Thermo Fisher Scientific). Recombinant proteins were purified from culture supernatants by affinity chromatography employing a Protein G column (Pierce, Thermo Fisher Scientific) with an ÄKTA FPLC system (GE Healthcare Europe, Freiburg, Germany) for IgG4 fusion proteins, and a Protein A column (Cytiva, Dreieich, Germany) with an ÄKTA Pure Protein Purification System (GE Healthcare) for IgG1 fusion proteins.

For immunizations, the Fc domains of IgG1-based hNKG2D-Fc and mNKG2D-Fc fusion proteins were removed by cleavage with TEV protease, processed Fc domains and unprocessed full-length proteins were removed by passing the reaction mixtures through a Protein A column, and remaining hNKG2D-His and mNKG2D-His fragments were purified using an immobilized metal affinity chromatography column (Cytiva) and a Strep-Tactin column (IBA Lifesciences, Göttingen, Germany). Immunization of a pathogen-free adult laying hen (*Gallus gallus domesticus*) was performed at Davids Biotechnologie GmbH (Regensburg, Germany), with 3 intramuscular injections of purified hNKG2D-His protein with AddaVax adjuvant (InvivoGen, Toulouse, France) at days 1, 14 and 28, followed by 2 booster injections with a 1:1 mix of hNKG2D-His and mNKG2D-His proteins at days 42 and 56. Peripheral blood was collected to confirm serum antibody reactivity with hNKG2D and mNKG2D by ELISA, and the animal was sacrificed at day 63 for spleen resection and subsequent RNA extraction.

### 2.3 Screening for NKG2D-binding scFv antibody fragments

An scFv yeast surface display (YSD) library was generated as described previously (33–35). Briefly, cDNA was synthesized from total splenic RNA, and VH and VL sequences were amplified in separate PCR reactions. Complete scFv sequences were then assembled from VH, (G_4_S)_3_ linker and VL fragments in a subsequent fusion PCR, and transferred into linearized YSD vector (pCT) via a homologous recombination-based process in *Saccharomyces cerevisiae* strain EBY100 (Thermo Fisher Scientific), following the yeast transformation protocol of Benatuil and colleagues for library generation (36). Prior to cell sorting, scFv expression and surface presentation was induced by inoculation of yeast cells in Synthetic Galactose minimal medium with Casein Amino Acids (SG-CAA) and incubation overnight at 30°C and 180 rpm.

General procedures for handling of yeast cells and library screening were described previously (34). Specifically, for screening of NKG2D binders, yeast cells were harvested by centrifugation, washed with PBS containing 0.1% (w/v) BSA (PBS-B), and incubated with human or murine NKG2D-Fc or NKG2D-His fusion proteins for 30 min on ice. After washing with PBS-B, surface display of the Myc-tag containing scFv antibodies and binding to recombinant NKG2D was detected simultaneously by incubation with a FITC-conjugated Myc-tag specific antibody (SH1-26E7.1.3; Miltenyi Biotec), and PE-conjugated anti-human IgG Fc (polyclonal) or AF647-conjugated His-tag-specific antibodies (4E3D10H2/E3) (both Thermo Fisher Scientific) for 20 min on ice in the dark. Then, cells were washed with PBS-B, and screened by flow cytometric cell sorting using a Sony SH800S device. Collected yeast cells were plated onto Synthetic Dextrose minimal medium with Casein Amino Acids (SD-CAA) agar plates and propagated for subsequent analysis or screening rounds by incubation at 30°C.

Four individual scFv antibody domains species cross-reactive with human and murine NKG2D (termed sc1, sc2, sc4 and sc7) were selected from the output of the library screening, and for subsequent analysis were linked to hinge, CH2 and CH3 domains of human IgG1 (UniProtKB: P01857-1, amino acid residues 99-330), expressed as Fc fusion proteins in Expi293F cells, and purified from culture supernatants via Protein A affinity chromatography as described above.

### 2.4 Design, expression and purification of bispecific killer cell engagers

Generation of a prototypic bispecific molecule (here termed hNKAB-ErbB2) consisting of an N-terminal scFv domain derived from human NKG2D-specific KYK-2.0 antibody, linked via a human IgG4 Fc region to a C-terminal scFv sequence specific for ErbB2 was described previously (18). For interaction with murine NKG2D, a similar mNKAB-ErbB2 molecule was generated by fusing the extracellular domain of murine NKG2D ligand MULT-1 (UniProtKB: D2CKI9, amino acid residues 26-211) to hinge, CH2 and CH3 domains of murine IgG1 (UniProtKB: P01868, amino acid residues 98-324, with the cysteine at position 102 replaced by a serine), followed by a (G_4_S)_2_ linker and the ErbB2-specific scFv(FRP5) antibody domain (37). Likewise, bispecific killer cell engagers employing the NKG2D-specific scFv antibody domains derived from the yeast display library screens were generated by replacing the KYK-2.0 scFv domain of hNKAB-ErbB2 with sc1, sc2, sc4 and sc7 sequences, yielding scNKAB-ErbB2(1), scNKAB-ErbB2(2), scNKAB-ErbB2(4) and scNKAB-ErbB2(7). All NKAB sequences were complemented by an N-terminal immunoglobulin heavy chain signal peptide, assembled *in silico*, *de novo* synthesized (GeneArt, Thermo Fisher Scientific), and inserted into mammalian expression vector pcDNA3. Expi293F cells were transiently transfected with the resulting plasmids, and recombinant molecules were purified from culture supernatants by affinity chromatography using either a Protein G column (hNKAB-ErbB2 and scNKAB-ErbB2 molecules) or a Protein A column (mNKAB-ErbB2) with an ÄKTA FPLC system as described above. Purity of recombinant NKAB molecules was confirmed by SDS-PAGE followed by Coomassie staining. NKAB-containing elution fractions were combined and dialyzed against DPBS. Protein concentrations were determined using a Nanodrop 1000 spectrophotometer (Thermo Fisher Scientific) considering molecular mass and extinction coefficient of the individual proteins.

### 2.5 Generation of NKG2D-CAR expressing NK cells and ErbB2-expressing tumor cells

The generation of established human NK-92 cells expressing an NKG2D-based chimeric antigen receptor that encompasses an immunoglobulin heavy chain signal peptide, the extracellular domain of human NKG2D, a (G_4_S)_2_ linker, a Myc-tag, a modified CD8α hinge region and transmembrane and intracellular signaling domains of human CD3ζ (hNKG2D.z, here termed hNKAR) was described previously (18, 20). A similar CAR based on murine NKG2D (mNKG2D.z, termed mNKAR) was designed by replacing the extracellular domain of human NKG2D with the corresponding murine sequence (UniProtKB: Q2TJJ6, amino acid residues 98-232, with the cysteine at position 99 replaced by a serine). The complete mNKAR sequence was then inserted into lentiviral transfer plasmid pHR’SIN-cPPT-SIRW upstream of IRES and near-infrared fluorescent protein (iRFP) sequences (38), resulting in vector pS-mNKAR-IRW. VSV-G pseudotyped vector particles were produced in HEK293T cells, and NK-92 cells were transduced as described previously (39). mNKAR-expressing NK-92 cells were enriched by sorting of iRFP-positive cells using a FACSAria Fusion Flow Cytometer (BD Biosciences, Heidelberg, Germany). Surface expression of NKG2D-CARs on hNKAR-NK-92 and mNKAR-NK-92 cells was confirmed by flow cytometry using an AF647-conjugated Myc-tag-specific antibody (9E10; BioLegend, Koblenz, Germany), and PE-conjugated antibodies specific for human NKG2D (BAT221; Miltenyi Biotec) or murine NKG2D (CX5; BioLegend). All flow cytometric measurements were performed with an LSRFortessa Cell Analyzer (BD Biosciences). Data were processed using FlowJo software (version 10.6.0; BD Biosciences). ErbB2-expressing CEM.NKR/ErbB2 and RMA/neo/ErbB2 cells were generated by transduction with VSV-G pseudotyped lentiviral vector encoding full-length human ErbB2 (20), followed by flow cytometric cell sorting using Alexa Fluor 647-conjugated anti-human ErbB2 antibody (24D2; BioLegend).

### 2.6 Degranulation of NK cells

Degranulation of NKAR-NK-92 cells upon exposure to immobilized anti-NKG2D scFv-Fc fusion proteins was analyzed by detecting surface expression of lysosomal-associated membrane protein 1 (LAMP-1, CD107a). The wells of a 96-well flat-bottom microtiter plate were coated overnight with 100 µl DPBS containing 500 ng of the respective protein. After washing the plate with DPBS and blocking unspecific binding sites with DPBS supplemented with 10% FCS for 20 min at room temperature, hNKAR-NK-92 and mNKAR-NK-92 cells were plated at 5x10^5^ cells/well in the presence of GolgiStop (BD Biosciences) and PE-conjugated CD107a-specific antibody (H4A3; BioLegend). NK cells kept in medium in the absence of scFv-Fc fusion proteins or stimulated with 50 ng/mL phorbol 12-myristate 13-acetate (PMA) and 500 ng/mL ionomycin (both Sigma-Aldrich) served as controls. After 4 hours of incubation at 37°C, the cells were washed with DPBS and analyzed by flow cytometry.

### 2.7 Binding assays

Binding of NKG2D-Fc or NKAB fusion proteins to tumor cells expressing ErbB2 or human or murine NKG2D ligands, as well as binding of NKAB molecules to NKAR-expressing NK-92 cells was investigated by flow cytometry. Cells were incubated with 12.5 nM of hNKG2D-Fc, mNKG2D-Fc or NKAB proteins followed by staining with APC-conjugated human or murine IgG-specific detection antibodies (both Jackson ImmunoResearch, Cambridgeshire, UK). Competition of soluble MICA by NKAB antibodies was analyzed by incubating hNKAR-NK-92 cells with 20 nM of recombinant His-tag-conjugated extracellular domain of MICA (SinoBiological, Eschborn, Germany) in the absence or presence of 5, 10 or 20 nM of a respective NKAB molecule. Remaining MICA bound to hNKAR-NK-92 cells was detected with APC-conjugated His-tag-specific antibody (J095G46; BioLegend). Simultaneous binding of NKAB antibodies to *ex vivo* expanded primary effector cells and the target antigen ErbB2 was analyzed by incubation of the cells with NKAB molecules, followed by staining with PE-conjugated recombinant extracellular domain of ErbB2 (AcroBiosystems, Basel, Switzerland). To discriminate different lymphocyte subsets, human cells were in addition stained with BV421-conjugated anti-CD56 (NCAM16.2; BD Biosciences), FITC-conjugated anti-CD3 (OKT3; BioLegend) and APC-conjugated anti-CD8 (BW135/80; Miltenyi Biotec) antibodies, and murine cells with BV421-conjugated anti-CD19 (6D5; BioLegend), APC-conjugated anti-NK1.1 (PK136; Miltenyi Biotec) and PE/Cyanine7-conjugated anti-CD3 (500A2; BioLegend) antibodies. NKG2D surface expression was investigated using PE-conjugated antibodies specific for human (BAT221; Miltenyi Biotec) or murine NKG2D (CX5; BioLegend). Dead cells were excluded by staining with fixable viability dye eFluor780 (eBioscience, Thermo Fisher Scientific). In all experiments, cells were blocked with human (Fc1; BD Biosciences) or murine (93; BioLegend) Fc receptor blocking agent prior to antibody staining.

### 2.8 Cytotoxicity assays

Cytotoxicity of NKAR-NK-92 cells and NKG2D-expressing primary human and murine lymphocytes was analyzed in flow cytometry-based assays as described previously (39). Briefly, tumor cells were labeled with Calcein Violet AM (CV) or Far Red (FR) (CellTrace; Invitrogen, Thermo Fisher Scientific) and incubated with effector cells at different effector to target cell (E/T) ratios for 3 hours (NKAR-NK-92 cells and human PBMCs) or 4 hours (murine splenocytes) at 37°C in the presence or absence of bispecific NKAB antibodies. Dead target cells were identified by staining with propidium iodide (PI) or 4’,6-diamidino-2-phenylindole (DAPI) followed by flow cytometric quantification of CV/PI, FR/PI or FR/DAPI double-positive cells with an LSRFortessa Cell Analyzer. Spontaneous target cell lysis was subtracted to calculate specific cytotoxicity. Data were analyzed using FlowJo software.

### 2.9 Statistical analysis

Unless stated otherwise, quantitative data are represented as mean with standard deviation (SD). Statistical significance (*P* value < 0.05) was determined using unpaired *t*-test. All statistical analyses were performed with Prism 10 (Version 10.1.1.323; GraphPad Software, Boston, MA).

## 3 Results

### 3.1 Generation of NKG2D-specific scFv antibodies

**Figure 1:**
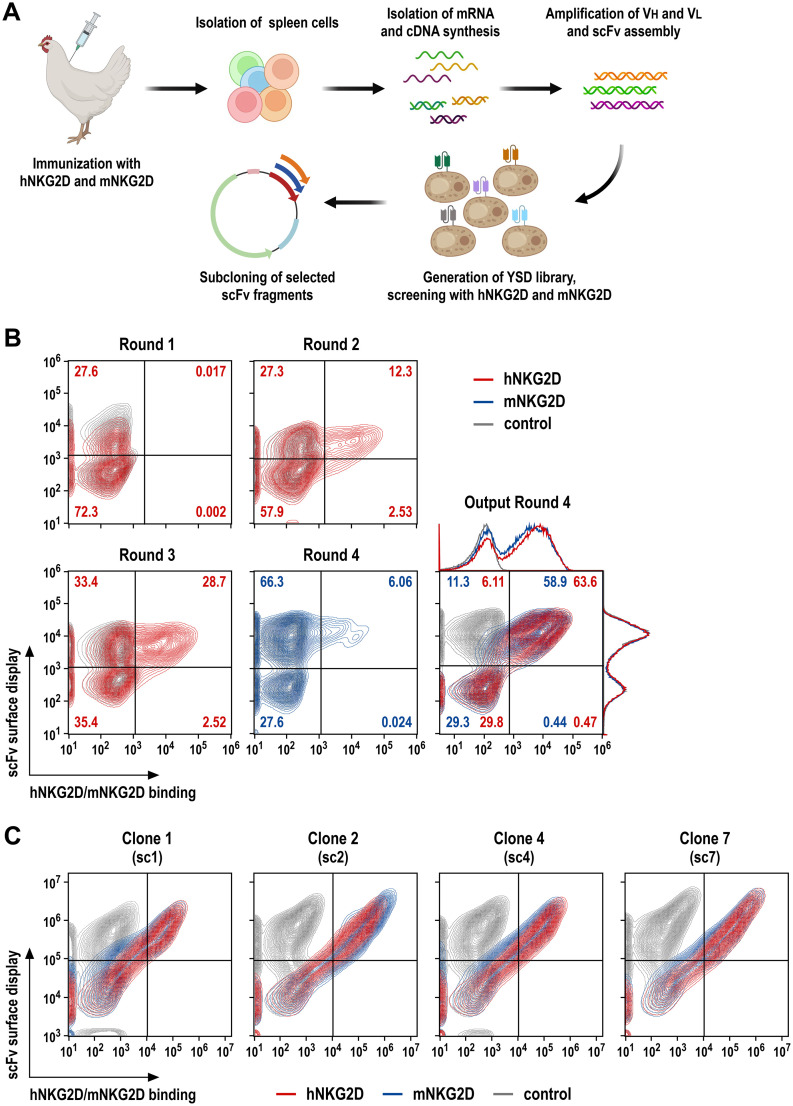
Screening of NKG2D-binding scFv antibody fragments by yeast surface display. **(A)** A laying hen was immunized with purified hNKG2D-His protein at days 1, 14 and 28, followed by booster injections with a 1:1 mix of hNKG2D-His and mNKG2D-His proteins at days 42 and 56. The spleen was resected at day 63 for RNA extraction and cDNA synthesis. VH and VL antibody sequences were amplified in separate PCR reactions, scFv sequences were assembled from VH, (G_4_S)_3_ linker and VL fragments in a fusion PCR, and transferred into a yeast surface display vector. **(B)** Yeast cells displaying NKG2D-binding scFv antibodies were identified by incubation with human NKG2D-Fc (screening rounds 1 and 2; red), or human (round 3; red) or murine NKG2D-His fusion proteins (round 4; blue), followed by PE-conjugated anti-human IgG Fc or AF647-conjugated His-tag-specific antibodies, respectively. ScFv surface display was confirmed by simultaneous staining with a FITC-conjugated antibody recognizing a C-terminal Myc-tag fused to the scFv sequences. In each case, yeast cells displaying NKG2D-binding scFv antibodies were enriched by flow cytometric cell sorting, expanded and subjected to a subsequent screening round. Cross-reactivity of displayed scFv antibodies of the yeast library obtained after the final screening round 4 was confirmed using hNKG2D-His and mNKG2D-His proteins. **(C)** Four individual yeast clones displaying species cross-reactive (sc) scFv antibodies (termed sc1, sc2, sc4 and sc7) binding to human and murine NKG2D as confirmed by staining with hNKG2D-His (red) and mNKG2D-His (blue) proteins were selected from the library screens for subsequent experiments. Yeast cells only incubated with Myc-tag-specific and secondary antibodies served as controls (gray).

Human and murine NKG2D share about 70% amino acid sequence identity in their extracellular domains. Hence, to ensure a sufficient immune response and generate antibodies reactive with human and murine receptors, we chose chicken for immunization as a phylogenetically distant species. Previous studies have demonstrated a satisfactory diversity of avian antibodies directed against epitopes conserved across mammalian species (33–35, 40). His-tagged recombinant proteins encompassing the extracellular domains of human (hNKG2D) and murine NKG2D (mNKG2D) were generated as described in Materials and Methods, and used for immunization of a pathogen-free adult laying hen with 3 intramuscular injections of hNKG2D-His at days 1, 14 and 28, followed by 2 booster injections with a 1:1 mixture of hNKG2D-His and mNKG2D-His proteins at days 42 and 56 (schematically shown in **Figure 1A**). Then, serum antibody reactivity with hNKG2D and mNKG2D was confirmed by ELISA (data not shown). The animal was sacrificed at day 63, and the spleen was resected for RNA extraction and cDNA preparation. Sequences encoding the variable domains of antibody heavy (VH) and light chains (VL) were amplified in separate PCR reactions, and randomly assembled into scFv antibodies with a (G_4_S)_3_ linker sequence connecting VH and VL domains following previously established procedures (33–35).

For screening of NKG2D binders, scFv sequences were then used to generate a yeast surface display (YSD) antibody library (34), comprising approximately 5 x 10^8^ transformants. To enrich for high affinity antibodies, the library was screened by 4 consecutive rounds of flow cytometric cell sorting with decreasing antigen concentrations, using recombinant hNKG2D-Fc fusion protein in rounds 1 (1000 nM) and 2 (500 nM), followed by screening with hNKG2D-His in round 3 (500 nM) and mNKG2D-His in round 4 (100 nM) (**Figure 1B**). This resulted in a yeast population with surface-displayed scFv molecules that demonstrated strong binding to the extracellular domains of both, human and murine NKG2D (Output Round 4). Ten single yeast cell clones were randomly selected, and analyzed by flow cytometry for binding of human and murine NKG2D, with four distinct clones displaying superior species cross-reactive (sc) binding to the human and murine receptors (clones sc1, sc2, sc4 and sc7) (**Figure 1C**).

### 3.2 Binding of NKG2D-specific scFv antibodies to NKG2D-CAR engineered NK cells

**Figure 2:**
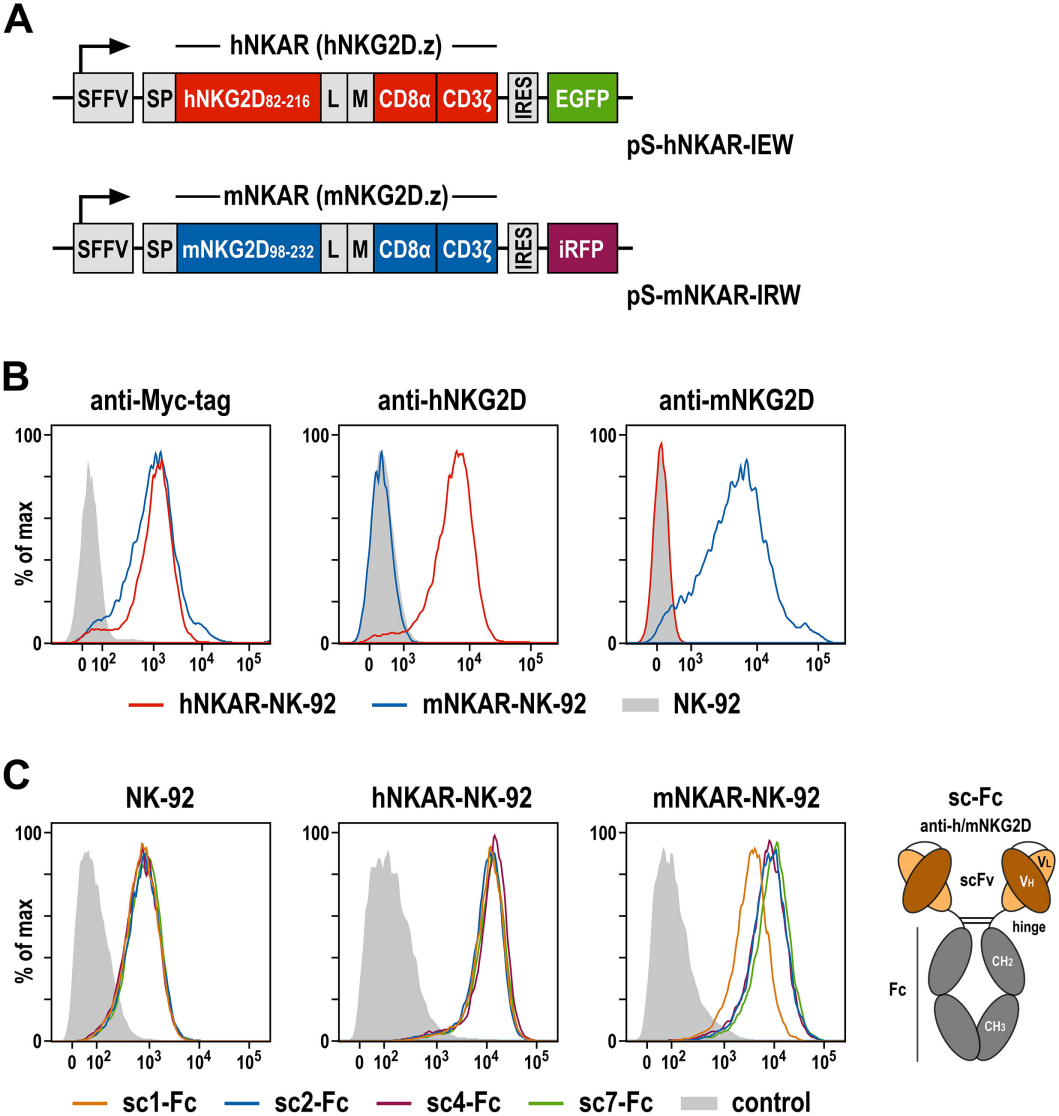
NKG2D-CAR expressing NK-92 cells as a model system to analyze NKG2D-binding antibodies. **(A)** Lentiviral transfer plasmids encoding NKG2D-based CARs under control of the Spleen Focus Forming Virus promoter (SFFV). hNKAR and mNKAR sequences consist of an immunoglobulin heavy chain signal peptide (SP), the extracellular domain of either human or murine NKG2D, a flexible (G_4_S)_2_ linker (L), a Myc-tag (M), a CD8α hinge region, and transmembrane and intracellular domains of CD3ζ. hNKAR and mNKAR sequences are followed by an internal ribosome entry site (IRES) and enhanced green fluorescent protein (EGFP) or near-infrared fluorescent protein (iRFP) cDNA, respectively. **(B)** Expression of NKG2D-CARs on the surface of sorted hNKAR-NK-92 and mNKAR-NK-92 cells was analyzed by flow cytometry as indicated using fluorochrome-labeled antibodies specific for the Myc-tag, human NKG2D or murine NKG2D. Parental NK-92 cells served as control. **(C)** Binding of scFv antibodies from the yeast surface display library screens to NKG2D and NKG2D-CAR expressing cells. The selected scFv antibodies sc1, sc2, sc4 and sc7 were expressed as scFv-Fc fusion proteins (sc-Fc; schematically shown on the right), and binding of the recombinant molecules to parental NK-92, hNKAR-NK-92 and mNKAR-NK-92 cells was analyzed by flow cytometry as indicated using an anti-human IgG antibody. Cells only stained with secondary antibody served as controls.

To functionally characterize the selected scFv antibodies, we employed derivatives of the clinically used human NK cell line NK-92 that were engineered to express NKG2D-based chimeric antigen receptors, either encompassing the extracellular domain of human or murine NKG2D, fused to a Myc-tag, a CD8α hinge region, and transmembrane and intracellular domains of CD3ζ (termed hNKAR and mNKAR, respectively) (**Figure 2A**). NK-92 cells expressing the human NKAR were previously shown to specifically recognize and kill human tumor cells which endogenously express NKG2D ligands, but not to target on their own murine cells only harboring murine NKG2DL (18, 20). To generate the similar mNKAR-NK-92 cells, a corresponding NKG2D-CD3ζ sequence employing the extracellular domain of murine NKG2D (amino acid residues 98-232, with an unpaired cysteine at position 99 replaced by a serine) was derived. Upon transduction with the respective lentiviral vector, NK-92 cells stably expressing the murine NKAR and an iRFP marker gene were enriched by flow cytometric cell sorting. Surface expression of hNKAR and mNKAR by the NK-92 derivatives was confirmed by staining with antibodies recognizing the Myc-tag contained in both CAR sequences, or selectively interacting with human or murine NKG2D (**Figure 2B**). As expected, hNKAR-NK-92 cells were unable to lyse murine A20 B-cell lymphoma and EL-4 T-cell lymphoma cells. Conversely, mNKAR-NK-92 cells effectively killed A20 cells which express murine NKG2DL, but not mNKG2DL-negative EL-4 cells, confirming functionality of the murine NKAR (see **Supplementary Figure 1**).

To analyze binding of the selected scFv antibodies derived from the immune library, recombinant scFv-Fc fusion proteins of clones sc1, sc2, sc4 and sc7 were generated and tested by flow cytometry using parental NK-92 cells and the hNKAR- or mNKAR-expressing derivatives (**Figure 2C**). Thereby all four antibodies displayed specific binding to NK-92 cells attributed to the moderate expression of endogenous NKG2D (18, 20), but markedly enhanced binding to hNKAR-NK-92 and mNKAR-NK-92 cells. This confirms specific and species cross-reactive interaction of the selected scFv antibodies with human and murine NKG2D presented on the surface of cytotoxic lymphocytes. Furthermore, if immobilized on plastic, the NKG2D-specific scFv-Fc fusion proteins also triggered degranulation of hNKAR-NK-92 and mNKAR-NK-92 cells, demonstrating their ability to activate human and murine NKG2D receptors (see **Supplementary Figure 2**).

### 3.3 Design of bispecific killer cell engagers recognizing NKG2D and the tumor-associated antigen ErbB2

**Figure 3:**
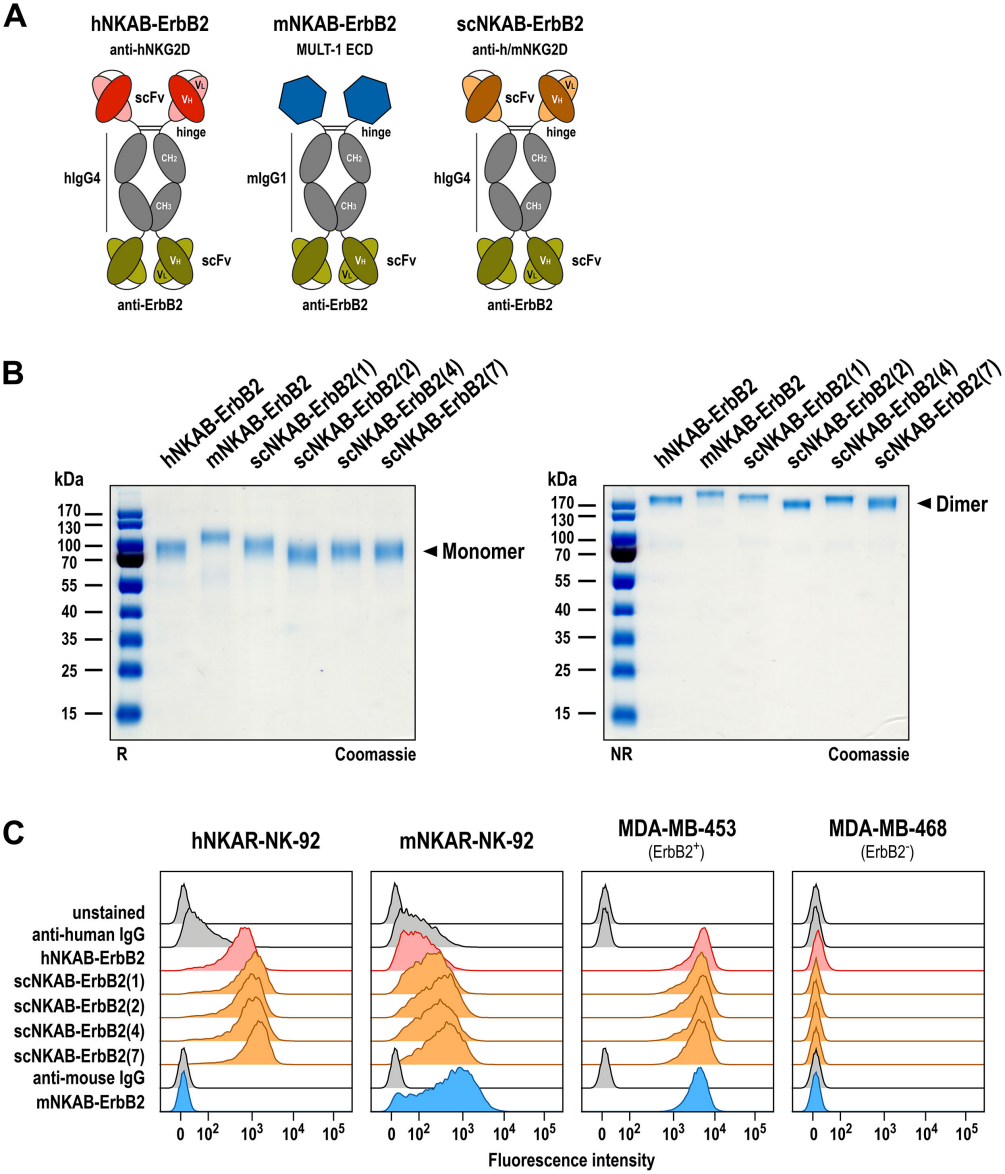
Generation of bispecific killer cell engagers. **(A)** Schematic representation of prototypic hNKAB-ErbB2 (left) and species cross-reactive scNKAB-ErbB2 molecules (right) that consist of an N-terminal scFv antibody fragment binding to NKG2D, followed by hinge, CH2 and CH3 domains of human IgG4 (hIgG4), a (G_4_S)_2_ linker, and an ErbB2-specific C-terminal scFv antibody fragment. mNKAB-ErbB2 (middle) carries the extracellular domain (ECD) of murine NKG2D ligand MULT-1 at the N-terminus, followed by hinge, CH2 and CH3 domains of murine IgG1 (mIgG1), a (G_4_S)_2_ linker, and an ErbB2-specific C-terminal scFv antibody fragment. Disulfide bridges facilitating formation of homodimers are indicated by lines. **(B)** Analysis of purified NKAB antibodies by SDS-PAGE under reducing (R, left) and non-reducing (NR, right) conditions and Coomassie staining. NKAB monomers and dimers are indicated by arrowheads. **(C)** Binding of purified NKAB molecules at a concentration of 12.5 nM to NK-92 cells expressing human (hNKAR-NK-92) or murine (mNKAR-NK-92) NKG2D-CARs, and ErbB2-positive MDA-MB-453 and ErbB2-negative MDA-MB-468 breast carcinoma cells was investigated by flow cytometry as indicated. Unstained cells and cells only incubated with secondary antibody served as controls.

Previously, we generated a prototypic bispecific antibody able to simultaneously interact with human NKG2D and the cellular proto-oncogene ErbB2 (HER2), which is overexpressed by a subtype of breast carcinomas and many other cancers of epithelial origin (41). This molecule, here termed hNKAB-ErbB2, consists of an N-terminal scFv moiety derived from antibody KYK-2.0 specific for human NKG2D (42), linked via the hinge, CH2 and CH3 regions of human IgG4 to a second, ErbB2-specific scFv domain derived from antibody FRP5 at the C-terminus (schematically shown in **Figure 3A**, left) (18, 37). Produced as a disulfide-linked homodimer, the hNKAB-ErbB2 molecule specifically redirected human lymphocytes endogenously expressing NKG2D or engineered with an NKG2D-based CAR to ErbB2-positive target cells irrespective of NKG2DL expression. Applying the same protein design, here we generated four similar bispecific killer cell engagers (termed scNKAB-ErbB2) that employ the selected sc1, sc2, sc4 and sc7 scFv moieties for NKG2D binding, but retain the IgG4 Fc domain and ErbB2-specific antibody fragment of the original hNKAB-ErbB2 molecule (**Figure 3A**, right). As a control protein, we also designed an mNKAB-ErbB2 molecule (**Figure 3A**, middle), which carries the extracellular domain of murine NKG2D ligand MULT-1 for selective binding to murine NKG2D, followed by hinge, CH2 and CH3 domains of murine IgG1, and the ErbB2-specific scFv fragment shared with the other NKAB molecules.

The NKAB antibodies were expressed as secreted proteins in transiently transfected Expi293F cells and purified from culture supernatants by Protein G (hNKAB-ErbB2, scNKAB-ErbB2 proteins) or Protein A (mNKAB-ErbB2) affinity chromatography. Elution fractions containing high amounts of recombinant proteins were combined and analyzed by SDS-PAGE under reducing and non-reducing conditions, followed by Coomassie staining to confirm integrity and purity of the recombinant proteins (**Figure 3B**). Thereby, purified hNKAB-ErbB2, mNKAB-ErbB2 and scNKAB-ErbB2 proteins predominantly consisted of intact disulfide-linked dimers (**Figure 3B**, right), which were separated into monomers under reducing conditions (**Figure 3B**, left). With a calculated molecular mass in monomeric form of 74 to 78 kDa, similar mobility of the NKAB proteins was expected in SDS gels under reducing conditions. Nevertheless, Coomassie staining revealed more pronounced differences in the apparent mass of the scNKAB-ErbB2 molecules, suggestive of different compactness of their tertiary structures. The murine reference molecule mNKAB-ErbB2 (calculated mass of the monomer: 74 kDa) even showed a major band at an apparent molecular mass of >100 kDa, most likely due to more pronounced N-linked glycosylation compared to the other NKAB proteins, as suggested by analysis with an N-linked glycosylation site prediction tool (43).

### 3.4 Binding of bispecific NKAB molecules to NKAR-expressing NK cells and ErbB2-positive tumor cells

Bispecific binding of the scNKAB-ErbB2 molecules was analyzed by flow cytometry using hNKAR-NK-92 and mNKAR-NK-92 cells, which as lymphocytes are negative for ErbB2, as well as ErbB2-overexpressing MDA-MB-453 and ErbB2-negative MDA-MB-468 breast carcinoma cells. hNKAB-ErbB2 and mNKAB-ErbB2 proteins were included for comparison. The results are shown in **Figure 3C**. NK-92 cells expressing the human NKG2D-CAR were strongly bound by hNKAB-ErbB2 and the four scNKAB-ErbB2 clones, but with the scNKAB-ErbB2 molecules displaying an approximately two-fold increase in median fluorescence intensity (MFI) when compared to the former, and scNKAB-ErbB2(7) showing the strongest binding (see **Supplementary Table 1**). As expected, mNKAB-ErbB2 did not bind to hNKAR-NK-92 cells, but via its MULT-1 ligand domain strongly interacted with the murine NKG2D-CAR of mNKAR-NK-92 cells. Due to the moderate endogenous expression of human NKG2D by NK-92 cells, prototypic hNKAB-ErbB2 also displayed limited binding to mNKAR-NK-92 cells. In contrast, confirming specific recognition of the murine NKG2D-CAR seen with the respective scFv-Fc fusion proteins, the species cross-reactive scNKAB-ErbB2 molecules interacted more strongly with mNKAR-NK-92 cells, with scNKAB-ErbB2(7) again displaying the most pronounced NKG2D interaction of the four clones (see **Supplementary Table 1**). scNKAB-ErbB2 molecules as well as hNKAB-ErbB2 and mNKAB-ErbB2 proteins showed comparable and very strong binding to ErbB2-positive MDA-MB-453 cells, but not to ErbB2-negative MDA-MB-468 cells, confirming that the ErbB2-specific FRP5 antibody domain shared by all NKAB molecules was functional to the same extent.

**Figure 4:**
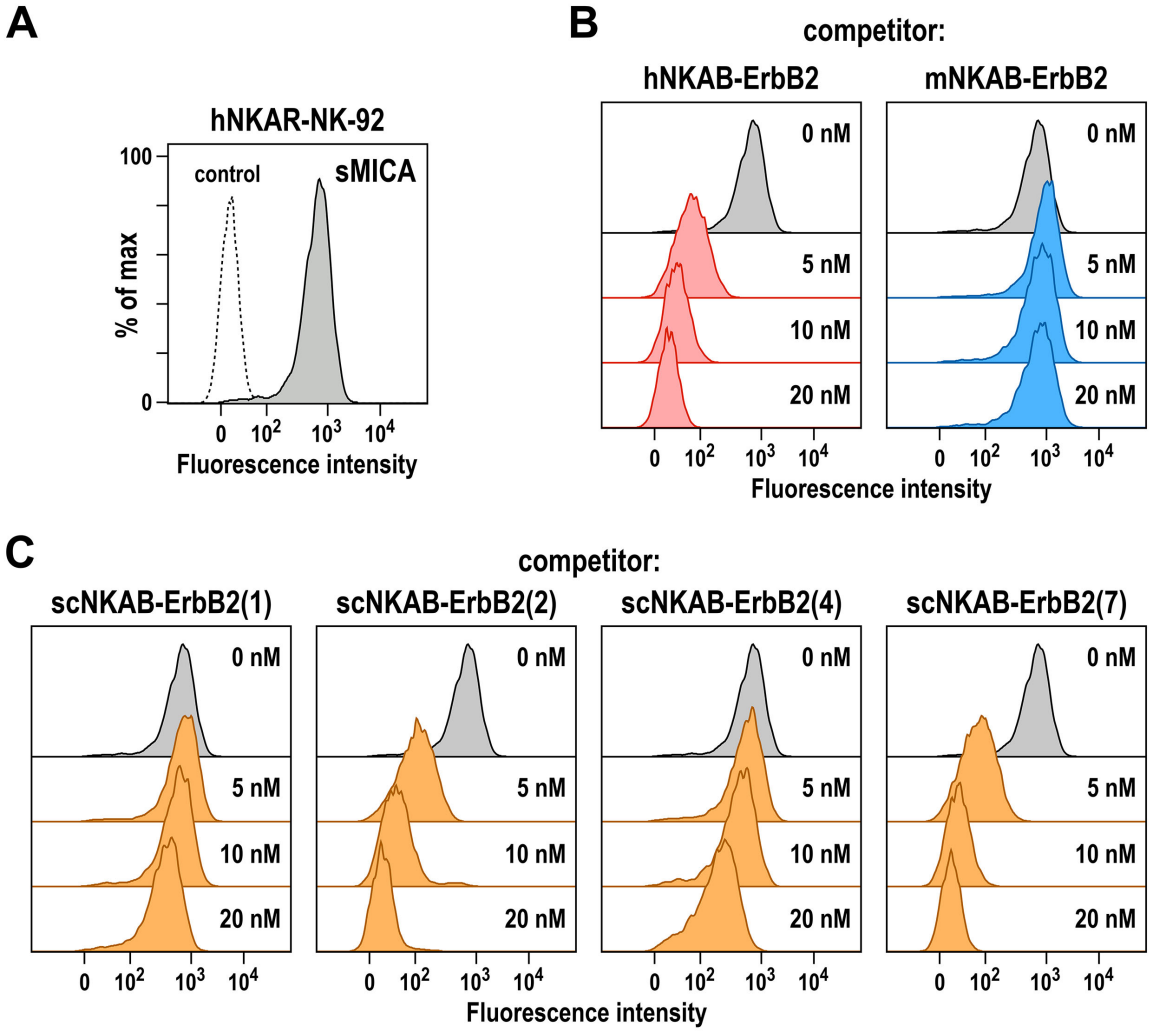
Competition of MICA binding by bispecific killer cell engagers. **(A)** Binding of His-tagged soluble MICA (sMICA) (20 nM) to hNKAR-NK-92 cells in the absence of NKAB molecules (gray area) was determined by flow cytometry with APC-conjugated anti-His-tag antibody. Control cells were only stained with secondary antibody (dashed line). **(B)** Competition of sMICA binding by prototypic hNKAB-ErbB2 was confirmed by incubation of cells with 20 nM of sMICA in the absence (gray area) or presence of increasing concentrations of hNKAB-ErbB2 (left). mNKAB-ErbB2 which is unable to interact with human NKG2D was included as negative control (right). **(C)** The ability of species cross-reactive NKAB antibodies to compete binding of sMICA to NKAR-NK-92 cells was investigated in similar experiments with purified scNKAB-ErbB2 proteins as indicated.

To test whether the epitopes of the generated scNKAB-ErbB2 molecules within NKG2D overlap with the binding site of a natural NKG2D ligand, we analyzed binding of 20 nM of recombinant soluble MICA (sMICA) to its cognate NKG2D and hNKAR receptors on hNKAR-NK-92 cells in the absence or presence of increasing concentrations of NKAB proteins ranging from 5 to 20 nM. In the absence of competitor, sMICA readily bound to hNKAR-NK-92 cells (**Figure 4A**). This was prevented by addition of prototypic hNKAB-ErbB2 known to compete with sMICA for NKG2D binding (18), but not by the mNKAB-ErbB2 control protein unable to interact with human NKG2D (**Figure 4B**). Similar to hNKAB-ErbB2, also species cross-reactive scNKAB-ErbB2(2) and scNKAB-ErbB2(7) molecules markedly inhibited sMICA binding, while scNKAB-ErbB2(1) did not compete sMICA, and scNKAB-ErbB2(4) only slightly reduced ligand binding to hNKAR-NK-92 cells at the highest concentration applied (**Figure 4C**), possibly caused by steric hindrance. Accordingly, the scFv antibody domains from clones sc2 and sc7 are competitive binders that can prevent access of soluble MICA, while scFv domains from clones sc1 and sc4 apparently bind to NKG2D regions distinct from the ligand binding site.

### 3.5 Simultaneous binding of NKAB molecules to NKG2D-positive primary lymphocytes and the ErbB2 target antigen

**Figure 5:**
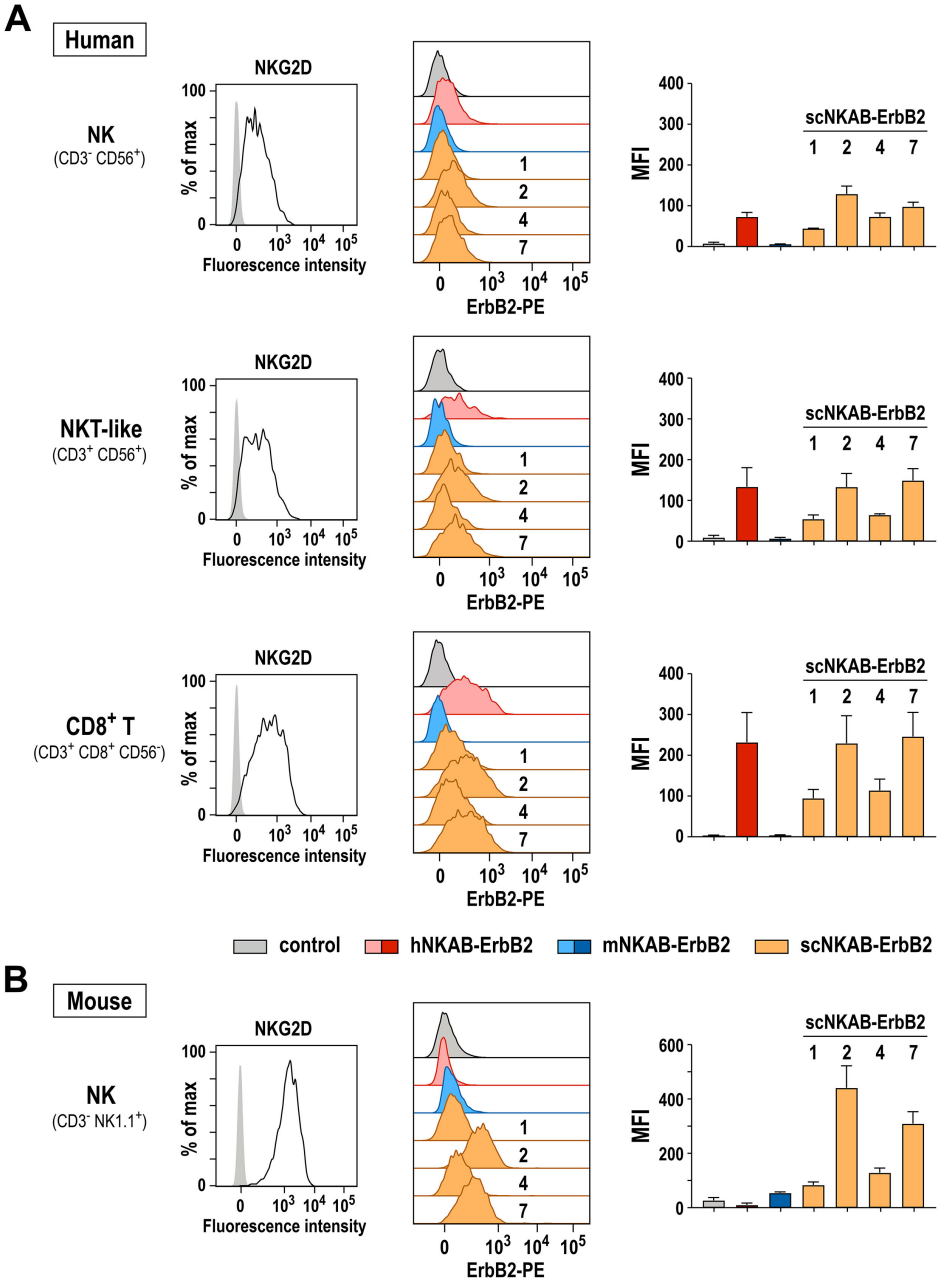
Bispecific binding of NKAB molecules to NKG2D-positive primary lymphocytes and ErbB2. Binding of purified scNKAB-ErbB2 proteins (orange), hNKAB-ErbB2 (red) and mNKAB-ErbB2 (blue) to gated NK, NKT-like and CD8^+^ T cell subpopulations of human PBMCs **(A)**, as well as *ex vivo* expanded murine NK cells **(B)** was analyzed by flow cytometry, detecting bound NKAB molecules with recombinant PE-conjugated ErbB2 extracellular domain to confirm simultaneous interaction with NKG2D and ErbB2. Cells stained with ErbB2-PE in the absence of an NKAB molecule (gray) served as controls. Middle panels show representative histograms of cells from one donor and one animal, respectively. Panels on the right display median fluorescence intensities (MFI). Mean values ± SD are shown; n=3 individual donors in **(A)** and n=2 individual animals in **(B)**. NKG2D surface expression by human NK, NKT-like and CD8^+^ T cell subpopulations and murine NK cells was confirmed by flow cytometry with NKG2D-specific antibodies (left panels).

Next, we performed binding assays to test whether the species cross-reactive scNKAB-ErbB2 proteins in addition to the artificial hNKAR and mNKAR receptors on NK-92 cells can also interact with primary human and murine lymphocytes endogenously expressing NKG2D in its native form. For human cells, freshly isolated PBMCs from three healthy donors were incubated with antibodies specific for CD3, CD56 and CD8 to differentiate between NK (CD3^-^ CD56^+^), NKT-like (CD3^+^ CD56^+^) and CD8-positive T cells (CD3^+^ CD8^+^ CD56^-^). In addition, cells were either incubated with an NKG2D-specific antibody to confirm NKG2D surface expression, or the different NKAB molecules to analyze their simultaneous interaction with NKG2D and the target antigen ErbB2. For this, binding of the NKAB proteins was detected with a PE-conjugated recombinant ErbB2 protein. As exemplarily shown for a representative donor in **Figure 5A**, left panels, gated NK, NKT-like and CD8^+^ T cell subpopulations were all strongly positive for NKG2D, with CD8^+^ T cells expressing even more elevated levels of the receptor than NK and NKT-like cells. Prototypic hNKAB-ErbB2 protein as well as all tested scNKAB-ErbB2 molecules readily bound to both, the NKG2D-positive primary lymphocytes and recombinant ErbB2, while mNKAB-ErbB2 as expected did not interact with the human cells (**Figure 5A**, middle). Thereby, in accordance with their enhanced NKG2D expression, CD8^+^ T cells were stained more strongly by the bispecific engagers. As indicated by the respective MFI values, prototypic hNKAB-ErbB2, scNKAB-ErbB2(2) and scNKAB-ErbB2(7) displayed most efficient binding to the NKG2D-positive human lymphocytes and recombinant ErbB2, with less pronounced signals obtained with scNKAB-ErbB2(1) and scNKAB-ErbB2(4) (**Figure 5A**, right).

Binding to murine lymphocytes endogenously expressing NKG2D was analyzed in similar experiments using *ex vivo* expanded murine NK cells obtained from two C57BL/6 mice (**Figure 5B**). Also in this case, scNKAB-ErbB2(2) and scNKAB-ErbB2(7) displayed the most efficient interaction with the NKG2D-positive cells and simultaneous binding to the ErbB2 target antigen. Specific signals obtained with mNKAB-ErbB2, scNKAB-ErbB2(1) and scNKAB-ErbB2(4) were much less pronounced, while hNKAB-ErbB2 in agreement with its selectivity for human NKG2D showed no binding to the murine cells.

### 3.6 NKAB-mediated redirection of primary lymphocytes to ErbB2-positive tumor cells

To investigate the influence of scNKAB-ErbB2 molecules on the antitumoral activity of primary human and murine lymphocytes, as model systems we first generated by lentiviral transduction and flow cytometric cell sorting stably ErbB2-expressing derivatives of the human and murine T lymphoblastic cell lines CEM.NKR and RMA/neo, which have both been described as largely resistant to the natural cytotoxicity of human and murine NK cells, respectively (44, 45). Specific binding of the four scNKAB-ErbB2 engagers and the hNKAB-ErbB2 and mNKAB-ErbB2 control molecules to the resulting CEM.NKR/ErbB2 and RMA/neo/ErbB2 cells was confirmed by flow cytometry, while no binding to the ErbB2-negative parental cell lines was detected (see **Supplementary Figure 3**).

**Figure 6:**
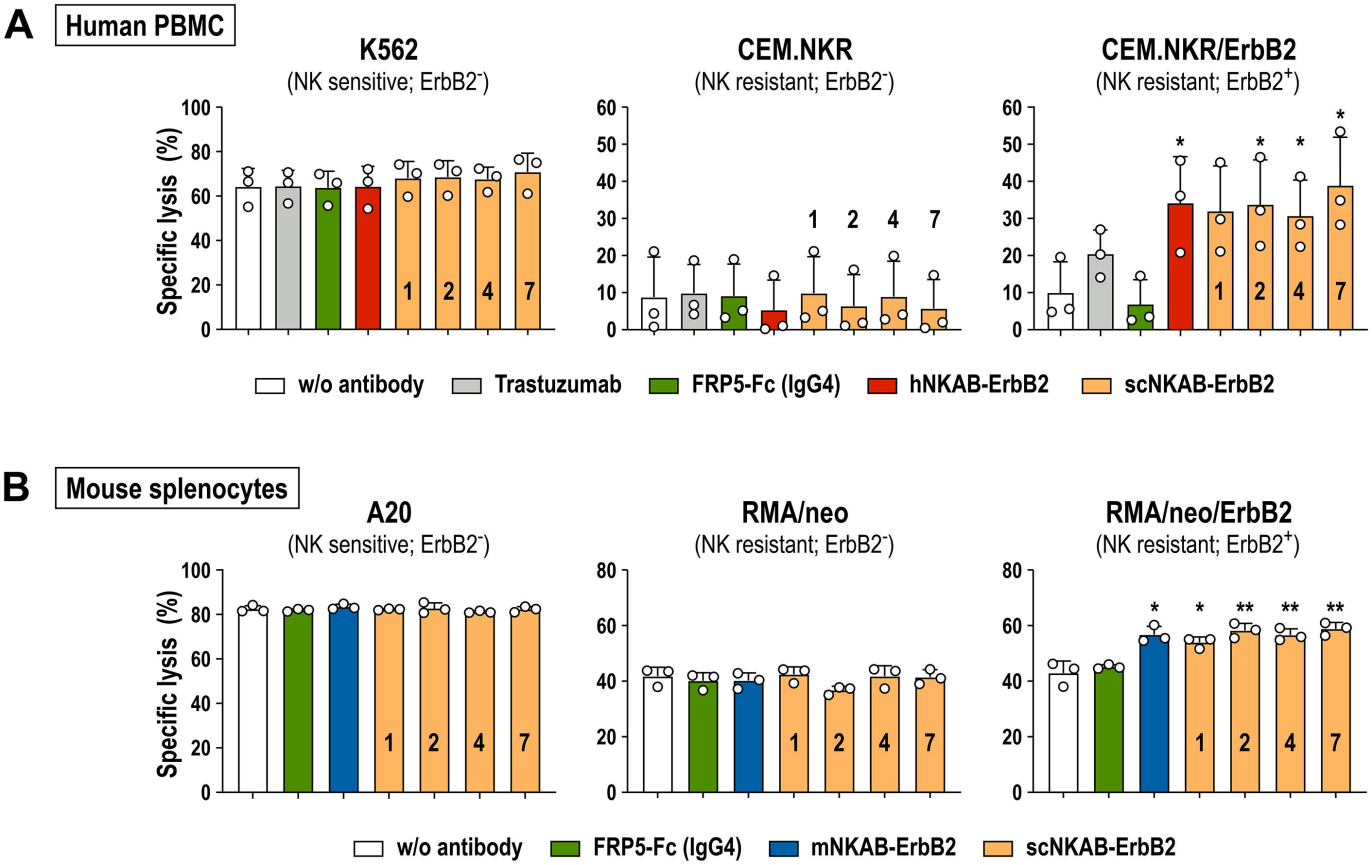
NKAB-mediated redirection of primary human and murine lymphocytes endogenously expressing NKG2D to ErbB2-expressing tumor cells. **(A)** Cytotoxicity of human PBMCs in the absence or presence of 0.64 nM of the indicated ErbB2-specific NKAB molecules against human K562 erythroleukemia, and CEM.NKR or CEM.NKR/ErbB2 T lymphoblastic cells was determined after 3 hours of co-incubation at an E/T ratio of 10:1. Samples kept in the absence of an antibody or incubated with 0.64 nM of trastuzumab or an ErbB2-specific scFv fusion protein containing a human IgG4 Fc domain (FRP5-Fc) were included as controls. **(B)** Cytotoxicity of murine splenocytes in the absence or presence of 0.64 nM of the indicated ErbB2-specific NKAB molecules against murine A20 B-cell lymphoma, and RMA/neo or RMA/neo/ErbB2 T lymphoblastic cells was determined after 4 hours of co-incubation at an E/T ratio of 20:1. Samples kept in the absence of an antibody or incubated with 0.64 nM of FRP5-Fc (IgG4) were included as controls. Mean values ± SD are shown; n=3 independent donors or animals. **, *p* < 0.01; *, *p* < 0.05. Statistical significance is indicated for differences in comparison to samples without antibody.

NKAB-mediated redirection of human lymphocytes to CEM.NKR/ErbB2 cells was then analyzed in cytotoxicity assays with PBMCs from three additional healthy donors, with gated NK, NKT-like and CD8^+^ T cell subpopulations consistently displaying high level expression of endogenous NKG2D (see **Supplementary Figure 4**). As expected, irrespective of the presence of NKAB-ErbB2 molecules, ErbB2-negative but NK-sensitive K562 erythroleukemia cells included as a positive control were readily killed by innate effector cells within the PBMCs (**Figure 6A**, left). In contrast, CEM.NKR/ErbB2 cells remained largely unaffected by PBMCs after 3 hours of co-incubation at an effector to target (E/T) ratio of 10:1, which was also the case in the presence of 0.64 nM of a scFv-Fc fusion protein (FRP5-Fc) containing the same ErbB2-specific scFv fragment and human IgG4 Fc domain as the scNKAB-ErbB2 molecules (46), but lacking NKG2D-specific binding (**Figure 6A**, right). Conversely, cytolytic activity of the PBMCs against the ErbB2-positive targets was enhanced by addition of the ErbB2-specific antibody trastuzumab, which in contrast to FRP5-Fc is of human IgG1 isotype and capable of inducing antibody-dependent cellular cytotoxicity (ADCC) by triggering FcγRIIIa (CD16a) on NK cells. However, most potent and significantly enhanced killing of CEM.NKR/ErbB2 by the PBMCs was observed in the presence of 0.64 nM of the prototypic hNKAB-ErbB2 protein and the four species cross-reactive scNKAB-ErbB2 engagers, while addition of these molecules had no effect on the low basal activity of PBMCs against ErbB2-negative CEM.NKR cells (**Figure 6A**, middle).

The influence of the scNKAB-ErbB2 molecules on cytotoxicity of primary murine lymphocytes was investigated using splenocytes from three C57BL/6 mice as effectors. Thereby, all cells of the gated NK and most cells of the NKT-like subpopulations displayed high level expression of endogenous NKG2D, while in contrast to human CD8^+^ T cells, only a small proportion of murine CD8^+^ T cells were NKG2D-positive (see **Supplementary Figure 5**). BALB/c-derived A20 B-cell lymphoma cells included as a positive control were readily killed by the C57BL/6 splenocytes after 4 hours of co-incubation at an E/T ratio of 20:1, without the presence of scNKAB-ErbB2 or mNKAB-ErbB2 engagers further enhancing cytotoxicity (**Figure 6B**, left). While ErbB2-negative RMA/neo cells with around 40% of specific lysis proved more sensitive toward the murine splenocytes than expected, also in this case addition of the scNKAB-ErbB2 or mNKAB-ErbB2 molecules had no significant effect on cell killing (**Figure 6B**, middle). This was different for RMA/neo/ErbB2 cells, which were more potently killed by splenocytes in the presence of 0.64 nM of the four scNKAB-ErbB2 engagers and mNKAB-ErbB2, while the FRP5-Fc isotype control molecule had no effect (**Figure 6B**, right).

### 3.7 Targeted cytotoxicity of NKG2D-CAR engineered effectors mediated by scNKAB-ErbB2 engagers

**Figure 7:**
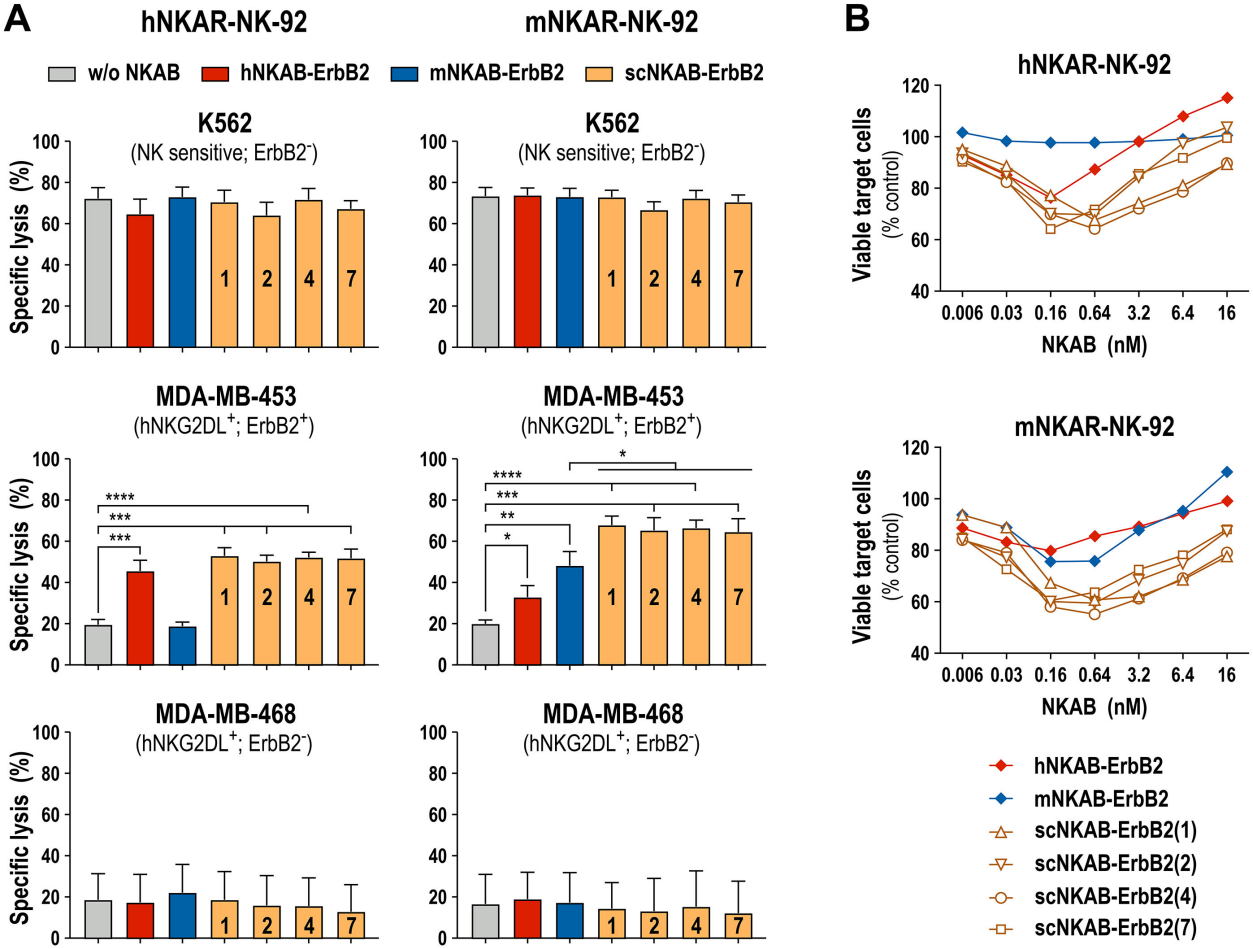
Redirection of NKG2D-CAR engineered NK-92 cells by ErbB2-targeted NKAB molecules. **(A)** NKAB-independent natural cytotoxicity of hNKAR-NK-92 (left panels) and mNKAR-NK-92 cells (right panels) against NK-sensitive K562 erythroleukemia cells (top) and NKAB-mediated killing of ErbB2-positive MDA-MB-453 breast carcinoma cells (middle) in the absence or presence of 0.64 nM of the indicated NKAB-ErbB2 molecules was determined after 3 hours of co-incubation at an E/T ratio of 5:1. ErbB2-negative MDA-MB-468 breast carcinoma cells (bottom) were included as control. Mean values ± SD are shown; n=3 independent experiments. ****, *p* < 0.0001; ***, *p* < 0.001; **, *p* < 0.01; *, *p* < 0.05. **(B)** Cytotoxic activity of hNKAR-NK-92 (top) or mNKAR-NK-92 cells (bottom) against MDA-MB-453 cells in the presence of increasing concentrations of the indicated NKAB-ErbB2 molecules was determined after 3 hours of co-incubation at an E/T ratio of 2:1. Data points represent mean values of the percentage of viable tumor cells normalized to values obtained after co-incubation of effector and target cells in the absence of NKAB antibodies. n=3 independent experiments.

To analyze the antitumoral activity of the scNKAB-ErbB2 molecules in combination with the NKG2D-CAR engineered effector cell lines hNKAR-NK-92 and mNKAR-NK-92, we employed human MDA-MB-453 breast cancer cells as targets which endogenously express different NKG2D ligands, but also high levels of ErbB2 (18). ErbB2-negative K562 erythroleukemia and MDA-MB-468 breast carcinoma cells were included as controls. After co-incubation for 3 hours at an E/T ratio of 5:1, NK-sensitive K562 cells were lysed effectively by hNKAR-NK-92 and mNKAR-NK-92 cells, which was not affected by the scNKAB-ErbB2 engagers, hNKAB-ErbB2 or mNKAB-ErbB2 (**Figure 7A**, top). While under the chosen conditions around 20% of ErbB2-positive MDA-MB-453 cells were already killed by hNKAR-NK-92 cells in the absence of NKAB antibodies, specific lysis was markedly enhanced to more than 50% by addition of 0.64 nM of each of the scNKAB-ErbB2 molecules (**Figure 7A**, middle). Also the prototypic hNKAB-ErbB2 molecule but not mNKAB-ErbB2 significantly increased cytotoxicity of NK-92 cells expressing the human NKG2D-CAR. In the case of mNKAR-NK-92 cells, the scNKAB-ErbB2 engagers were most potent in enhancing targeted cytotoxicity against MDA-MB-453 cells, with specific lysis even more pronounced than when combined with hNKAR-NK-92 cells. Likewise, mNKAB-ErbB2 significantly triggered the murine NKG2D-CAR against the ErbB2-positive target cells. Also moderate but statistically significant activity of hNKAB-ErbB2 was detected, which cannot engage the mNKAR, but the endogenous human NKG2D of mNKAR-NK-92 cells. In the case of MDA-MB-468 breast carcinoma cells which are negative for ErbB2, basal cytotoxic activity of hNKAR-NK-92 and mNKAR-NK-92 cells was not affected by any of the ErbB2-specific NKAB molecules (**Figure 7A**, bottom).

For hNKAB-ErbB2, concentrations between 0.16 and 0.64 nM were previously established as optimal to trigger effective cytotoxicity of NKG2D-positive primary lymphocytes and hNKAR-NK-92 cells. Concentrations lower than that were insufficient to fully activate the effector cells, and higher engager concentrations led to competition of productive interactions with the target receptors by free protein, both resulting in gradually reduced cytotoxicity (18). To test whether this is also the case for the newly developed scNKAB-ErbB2 molecules, in the next set of experiments hNKAR-NK-92 and mNKAR-NK-92 cells were co-incubated with ErbB2-positive MDA-MB-453 target cells at an E/T ratio of 2:1 in the presence of increasing concentrations of the different engagers, ranging from 0.006 to 16 nM (**Figure 7B**). Under these conditions, prototypic hNKAB-ErbB2 was most active at a concentration of 0.16 nM in reducing the number of viable target cells by hNKAR-NK-92, and triggered by endogenous human NKG2D, by mNKAR-NK-92 cells. The mNKAB-ErbB2 control protein had most pronounced activity in combination with mNKAR-NK-92 cells at concentrations of 0.16 and 0.64 nM, but confirming the results described above, was inactive in combination with hNKAR-NK-92 cells. The four scNKAB-ErbB2 engagers again showed much more pronounced activity in combination with hNKAR-NK-92 and mNKAR-NK-92 cells than hNKAB-ErbB2 and mNKAB-ErbB2, with 0.16 and 0.64 nM identified as optimal antibody concentrations. Importantly, even under suboptimal conditions, the species cross-reactive molecules retained higher activity than hNKAB-ErbB2 and mNKAB-ErbB2 at their optimal concentrations, suggesting more stable and productive immunological synapse formation over a wide range of scNKAB-ErbB2 concentrations.

## 4 Discussion

The activating receptor NKG2D and its ligands represent an important system to sense cellular stress upon malignant transformation or infection by pathogens, and enable innate lymphocytes and subsets of T cells to selectively and efficiently eliminate the affected target cells (8, 9). Different approaches have been developed to employ this mechanism for cancer immunotherapy, which include boosting NKG2DL-induced activation of effector lymphocytes with NKG2D-based CARs, pharmacological enhancement of NKG2DL expression on cancer cells, preventing proteolytic ligand shedding, and designing BiKE molecules that redirect cytotoxic effectors to tumor cells independent from NKG2DL recognition (2, 7, 12, 47, 48). In our study, we generated four novel bispecific killer cell engagers that displayed cross-reactive binding to human and murine NKG2D receptors, and selectively and efficiently redirected NKG2D-positive primary human and murine lymphocytes as well as NK cells engineered with human or murine NKG2D-CARs to cancer cells expressing the clinically highly relevant tumor-associated antigen ErbB2 (HER2).

Formation of species cross-reactive NKG2D antibodies was induced by consecutive immunization of a chicken with recombinant human and murine NKG2D proteins, and the most effective binders were then selected by screening of a scFv antibody yeast surface display library using decreasing concentrations of the human and murine antigens (33–35, 40). Binding and degranulation experiments with recombinant scFv-Fc fusion proteins and NK-92 cells expressing NKG2D-CARs derived from human (hNKAR) or murine NKG2D (mNKAR) confirmed specificity of the selected antibody clones sc1, sc2, sc4 and sc7, and demonstrated their ability to activate both, human and murine NKG2D receptors (see **Supplementary Figure 2**). Following the validated design of an ErbB2-specific NKG2D engager that exclusively interacts with human NKG2D (here termed hNKAB-ErbB2) (18, 42), we generated four similar scNKAB-ErbB2 molecules which all carry an N-terminal NKG2D-binding domain, connected by a human IgG Fc region to the ErbB2-binding domain at the C-terminus. While NKAB molecules based on the structure of ADCC-inducing IgG1 are functional (18), here we chose IgG4 to limit simultaneous interaction with CD16a and to clearly attribute the observed effects to NKG2D engagement (49). Facilitated by disulfide bridges within the IgG hinge region, the scNKAB-ErbB2 molecules were expressed as tetravalent homodimers, which readily interacted with hNKAR-NK-92 and mNKAR-NK-92 cells as well as ErbB2-expressing breast carcinoma cells. Thereby, all four scNKAB-ErbB2 molecules bound more effectively to the human NKG2D receptor than the prototypic hNKAB-ErbB2 molecule, with scNKAB-ErbB2(7) displaying the strongest interaction (see **Supplementary Table 1**).

In competition assays with an excess of soluble MICA, scNKAB-ErbB2(2) and scNKAB-ErbB2(7) proteins effectively prevented binding of the natural ligand to NKG2D, while scNKAB-ErbB2(1) and scNKAB-ErbB2(4) did not compete with sMICA. This is indicative of the epitopes of the latter being distinct from the ligand binding site. Although we only investigated blockade of MICA, this likely extends to other human NKG2DL, which share a similar binding surface on NKG2D (50, 51). Interestingly, when simultaneous interaction with NKG2D-expressing PBMC subpopulations and the ErbB2 target antigen was investigated by detecting bound NKAB molecules with recombinant ErbB2, prototypic hNKAB-ErbB2 as well as scNKAB-ErbB2(2) and scNKAB-ErbB2(7), which all compete with MICA, resulted in stronger signals. Likewise, scNKAB-ErbB2(2) and scNKAB-ErbB2(7) displayed more pronounced bispecific binding to NKG2D-positive murine NK cells and recombinant ErbB2. Since this differential binding did not affect lymphocyte activation in cytotoxicity assays with tumor cells, it may reflect different accessibility of the ErbB2-specific scFv domain of NKG2D-bound NKAB molecules for soluble ErbB2 monomers, without obvious impact on the higher avidity interactions of the homodimeric NKAB molecules with naturally expressed ErbB2 that is anchored in the target cell membrane.

Proteolytic shedding of NKG2D ligands has been described as a mechanism of tumor cells to evade NKG2D-mediated immune surveillance, with the released soluble NKG2DL not only reducing ligand density on the target cell surface, but also impairing immune responses by blockade and downregulation of NKG2D on cytolytic effector cells (13, 14). While non-competing BiKE molecules similar to scNKAB-ErbB2(1) and scNKAB-ErbB2(4) were shown to remain functional in the presence of NKG2D ligands (17), the same was demonstrated for MICA-competing prototypic hNKAB-ErbB2 (18). Enhanced by avidity effects of the bivalent NKG2D binder, hNKAB-ErbB2 prevented occupation of NKG2D by soluble MICA even at high concentrations, and restored NKG2D-dependent effector cell activity (18). This could also be expected for the NKG2DL-competing molecules scNKAB-ErbB2(2) and scNKAB-ErbB2(7). Indeed, despite quantitative differences in their binding to NKG2D (see **Supplementary Table 1**), ligand-competing and non-competing scNKAB-ErbB2 molecules were equally effective in redirecting primary lymphocytes and NKG2D-CAR expressing NK cells to ErbB2-positive tumor targets.

With a structure and molecular mass similar to an intact IgG antibody, Fc-containing scNKAB-ErbB2 molecules are expected to have a longer serum half-life than small tandem scFv-scFv fusions like blinatumomab, which requires continuous infusion in patients to achieve relevant concentrations in the blood (52). Furthermore, bivalent binding of the homodimeric NKAB proteins to both of their targets, ErbB2 and NKG2D, is likely important for formation of a more stable immunological synapse and effective tumor cell killing, as indicated by data from our previous study with prototypic hNKAB-ErbB2. When dimer formation of hNKAB-ErbB2 was prevented by removal of disulfide bridges within the antibody hinge region, the resulting monomer was less efficient in triggering activation of NKG2D-CAR expressing NK cells against ErbB2-positive cancer cells (18). Likewise, in the case of nanobody-based bispecific NKG2DxErbB2 antibodies, bivalent binders displayed a 20- to 60-fold increase in affinity when compared to their monovalent counterparts (17). When tested at a fixed protein concentration of 0.64 nM in combination with primary human and murine lymphocytes as effectors, the species cross-reactive scNKAB-ErbB2 molecules showed similar antitumoral activity as hNKAB-ErbB2 and mNKAB-ErbB2, respectively. Nevertheless, more detailed analysis with hNKAR-NK-92 and mNKAR-NK-92 cells carrying CARs based on human and murine NKG2D, despite a similar optimum at 0.16 to 0.64 nM, revealed marked scNKAB-ErbB2 activity over a broader concentration range than hNKAB-ErbB2 and mNKAB-ErbB2. In particular scNKAB-ErbB2(1) and scNKAB-ErbB2(4) showed less pronounced reduction of effector cell activity at higher NKAB concentrations due to competition with free protein, suggesting more stable cell-cell contacts mediated by these molecules.

To the best of our knowledge, this is the first report on bispecific killer cell engagers that recruit both, human and murine lymphocytes. We devised a strategy for immunization and screening, which despite differences in the amino acid sequences of the human and murine antigens, facilitated the generation of species cross-reactive NKG2D binders. Similar to studies with antibodies that target immune checkpoint molecules like PD-1, CTLA-4 and TIGIT (53–55), this could aid further preclinical development of the bispecific killer cell engagers by allowing testing in immunocompetent mouse tumor models. Thereby, investigating their interaction with endogenous immune cells *in vivo* as done here with isolated murine splenocytes *ex vivo*, and evaluating potential adverse effects may provide insights not possible in more artificial tumor xenograft models in immunodeficient mice. The ErbB2-specific FRP5 antibody domain used in our study does not react with the murine ErbB2 homolog (56). Nevertheless, the high degree of sequence identity between the human and murine antigens still allows the evaluation of ErbB2-targeted immunotherapies with murine tumor cells modified to express human ErbB2 in immunocompetent BALB/c and C57BL/6 mouse models (18, 57). Our data demonstrate enhanced functionality of the newly generated scNKAB-ErbB2 engagers compared to the previously described species-restricted hNKAB-ErbB2 molecule. Thereby, the bispecific scNKAB antibodies proved effective in specifically redirecting the cytotoxic activity of primary lymphocytes as well as NKG2D-CAR engineered NK cells to ErbB2-positive cancer targets. Due to their modular design, these molecules could easily be adapted to interact with CD16a in addition to NKG2D and to target other tumor-associated surface antigens by exchanging their respective Fc and scFv domains (18, 20). At present, the generated NKG2D-binding moieties are still of avian origin. Hence, an important next step for further development will be the humanization of their sequences following established procedures (58).

## Data Availability

The original contributions presented in the study are included in the article/**Supplementary Material**. Further inquiries can be directed to the corresponding authors.

## References

[1] Goebeler ME Bargou RC. T cell-engaging therapies - BiTEs and beyond. Nat Rev Clin Oncol. (2020) 17:418–34. doi: 10.1038/s41571-020-0347-5 32242094

[2] Peipp M Klausz K Boje AS Zeller T Zielonka S Kellner C. Immunotherapeutic targeting of activating natural killer cell receptors and their ligands in cancer. Clin Exp Immunol. (2022) 209:22–32. doi: 10.1093/cei/uxac028 35325068 PMC9307233

[3] Klein C Brinkmann U Reichert JM Kontermann RE. The present and future of bispecific antibodies for cancer therapy. Nat Rev Drug Discovery. (2024) 23:301–19. doi: 10.1038/s41573-024-00896-6 38448606

[4] Fenis A Demaria O Gauthier L Vivier E Narni-Mancinelli E. New immune cell engagers for cancer immunotherapy. Nat Rev Immunol. (2024) 7:471–86. doi: 10.1038/s41577-023-00982-7 38273127

[5] Rolin C Zimmer J Seguin-Devaux C. Bridging the gap with multispecific immune cell engagers in cancer and infectious diseases. Cell Mol Immunol. (2024) 21:643–61. doi: 10.1038/s41423-024-01176-4 PMC11214628 38789528

[6] Gauthier L Virone-Oddos A Beninga J Rossi B Nicolazzi C Amara C. Control of acute myeloid leukemia by a trifunctional NKp46-CD16a-NK cell engager targeting CD123. Nat Biotechnol. (2023) 41:1296–306. doi: 10.1038/s41587-022-01626-2 PMC10497414 36635380

[7] Lazarova M Wels WS Steinle A. Arming cytotoxic lymphocytes for cancer immunotherapy by means of the NKG2D/NKG2D-ligand system. Expert Opin Biol Ther. (2020) 20:1491–501. doi: 10.1080/14712598.2020.1803273 32726145

[8] Stojanovic A Correia MP Cerwenka A. The NKG2D/NKG2DL axis in the crosstalk between lymphoid and myeloid cells in health and disease. Front Immunol. (2018) 9:827. doi: 10.3389/fimmu.2018.00827 29740438 PMC5924773

[9] Wensveen FM Jelenčić V Polić B. NKG2D: A master regulator of immune cell responsiveness. Front Immunol. (2018) 9:441. doi: 10.3389/fimmu.2018.00441 29568297 PMC5852076

[10] Cerwenka A Lanier LL. NKG2D ligands: unconventional MHC class I-like molecules exploited by viruses and cancer. Tissue Antigens. (2003) 61:335–43. doi: 10.1034/j.1399-0039.2003.00070.x 12753652

[11] Raulet DH Gasser S Gowen BG Deng W Jung H. Regulation of ligands for the NKG2D activating receptor. Annu Rev Immunol. (2013) 31:413–41. doi: 10.1146/annurev-immunol-032712-095951 PMC4244079 23298206

[12] Paczulla AM Rothfelder K Raffel S Konantz M Steinbacher J Wang H. Absence of NKG2D ligands defines leukaemia stem cells and mediates their immune evasion. Nature. (2019) 572:254–9. doi: 10.1038/s41586-019-1410-1 PMC6934414 31316209

[13] Salih HR Rammensee HG Steinle A. Cutting edge: down-regulation of MICA on human tumors by proteolytic shedding. J Immunol. (2002) 169:4098–102. doi: 10.4049/jimmunol.169.8.4098 12370336

[14] Groh V Wu J Yee C Spies T. Tumour-derived soluble MIC ligands impair expression of NKG2D and T-cell activation. Nature. (2002) 419:734–8. doi: 10.1038/nature01112 12384702

[15] Song H Kim J Cosman D Choi I. Soluble ULBP suppresses natural killer cell activity via down-regulating NKG2D expression. Cell Immunol. (2006) 239:22–30. doi: 10.1016/j.cellimm.2006.03.002 16630603

[16] Chan WK Kang S Youssef Y Glankler EN Barrett ER Carter AM. A CS1-NKG2D bispecific antibody collectively activates cytolytic immune cells against multiple myeloma. Cancer Immunol Res. (2018) 6:776–87. doi: 10.1158/2326-6066.CIR-17-0649 PMC6030494 29769244

[17] Raynaud A Desrumeaux K Vidard L Termine E Baty D Chames P. Anti-NKG2D single domain-based antibodies for the modulation of anti-tumor immune response. Oncoimmunology. (2020) 10:1854529. doi: 10.1080/2162402X.2020.1854529 33457075 PMC7781768

[18] Zhang C Röder J Scherer A Bodden M Pfeifer Serrahima J Bhatti A. Bispecific antibody-mediated redirection of NKG2D-CAR natural killer cells facilitates dual targeting and enhances antitumor activity. J Immunother Cancer. (2021) 9:e002980. doi: 10.1136/jitc-2021-002980 34599028 PMC8488744

[19] Lutz S Klausz K Albici AM Ebinger L Sellmer L Teipel H. Novel NKG2D-directed bispecific antibodies enhance antibody-mediated killing of Malignant B cells by NK cells and T cells. Front Immunol. (2023) 14:1227572. doi: 10.3389/fimmu.2023.1227572 37965326 PMC10641740

[20] Kiefer A Prüfer M Röder J Pfeifer Serrahima J Bodden M Kühnel I. Dual targeting of glioblastoma cells with bispecific killer cell engagers directed to EGFR and ErbB2 (HER2) facilitates effective elimination by NKG2D-CAR-engineered NK cells. Cells. (2024) 13:246. doi: 10.3390/cells13030246 38334638 PMC10854564

[21] von Strandmann EP Hansen HP Reiners KS Schnell R Borchmann P Merkert S. A novel bispecific protein (ULBP2-BB4) targeting the NKG2D receptor on natural killer (NK) cells and CD138 activates NK cells and has potent antitumor activity against human multiple myeloma *in vitro* and *in vivo* . Blood. (2006) 107:1955–62. doi: 10.1182/blood-2005-05-2177 16210338

[22] Rothe A Jachimowicz RD Borchmann S Madlener M Keßler J Reiners KS. The bispecific immunoligand ULBP2-aCEA redirects natural killer cells to tumor cells and reveals potent anti-tumor activity against colon carcinoma. Int J Cancer. (2014) 134:2829–40. doi: 10.1002/ijc.28609 24242212

[23] Kellner C Günther A Humpe A Repp R Klausz K Derer S. Enhancing natural killer cell-mediated lysis of lymphoma cells by combining therapeutic antibodies with CD20-specific immunoligands engaging NKG2D or NKp30. Oncoimmunology. (2016) 5:e1058459. doi: 10.1080/2162402X.2015.1058459 26942070 PMC4760288

[24] Pan M Wang F Nan L Yang S Qi J Xie J. αVEGFR2-MICA fusion antibodies enhance immunotherapy effect and synergize with PD-1 blockade. Cancer Immunol Immunother. (2023) 72:969–84. doi: 10.1007/s00262-022-03306-1 PMC10991987 36227341

[25] Miller JS Lanier LL. Natural killer cells in cancer immunotherapy. Annu Rev Cancer Biol. (2019) 3:77–103. doi: 10.1146/annurev-cancerbio-030518-055653

[26] Maskalenko NA Zhigarev D Campbell KS. Harnessing natural killer cells for cancer immunotherapy: dispatching the first responders. Nat Rev Drug Discovery. (2022) 21:559–77. doi: 10.1038/s41573-022-00413-7 PMC10019065 35314852

[27] Arai S Meagher R Swearingen M Myint H Rich E Martinson J. Infusion of the allogeneic cell line NK-92 in patients with advanced renal cell cancer or melanoma: a phase I trial. Cytotherapy. (2008) 10:625–32. doi: 10.1080/14653240802301872 18836917

[28] Tonn T Schwabe D Klingemann HG Becker S Esser R Koehl U. Treatment of patients with advanced cancer with the natural killer cell line NK-92. Cytotherapy. (2013) 15:1563–70. doi: 10.1016/j.jcyt.2013.06.017 24094496

[29] Tang X Yang L Li Z Nalin AP Dai H Xu T. First-in-man clinical trial of CAR NK-92 cells: safety test of CD33-CAR NK-92 cells in patients with relapsed and refractory acute myeloid leukemia. Am J Cancer Res. (2018) 8:1083–9. PMC6048396 30034945

[30] Burger MC Forster MT Romanski A Straßheimer F Macas J Zeiner PS. Intracranial injection of natural killer cells engineered with a HER2-targeted chimeric antigen receptor in patients with recurrent glioblastoma. Neuro Oncol. (2023) 25:2058–71. doi: 10.1093/neuonc/noad087 PMC10628939 37148198

[31] Kerbauy LN Marin ND Kaplan M Banerjee PP Berrien-Elliott MM Becker-Hapak M. Combining AFM13, a bispecific CD30/CD16 antibody, with cytokine-activated blood and cord blood-derived NK cells facilitates CAR-like responses against CD30(+) Malignancies. Clin Cancer Res. (2021) 27:3744–56. doi: 10.1158/1078-0432.CCR-21-0164 PMC8254785 33986022

[32] Gong JH Maki G Klingemann HG. Characterization of a human cell line (NK-92) with phenotypical and functional characteristics of activated natural killer cells. Leukemia. (1994) 8:652–8. 8152260

[33] Grzeschik J Yanakieva D Roth L Krah S Hinz SC Elter A. Yeast surface display in combination with fluorescence-activated cell sorting enables the rapid isolation of antibody fragments derived from immunized chickens. Biotechnol J. (2019) 14:e1800466. doi: 10.1002/biot.201800466 30350923

[34] Bogen JP Grzeschik J Krah S Zielonka S Kolmar H. Rapid generation of chicken immune libraries for yeast surface display. Methods Mol Biol. (2020) 2070:289–302. doi: 10.1007/978-1-4939-9853-1_16 31625102

[35] Schoenfeld K Harwardt J Habermann J Elter A Kolmar H. Conditional activation of an anti-IgM antibody-drug conjugate for precise B cell lymphoma targeting. Front Immunol. (2023) 14:1258700. doi: 10.3389/fimmu.2023.1258700 37841262 PMC10569071

[36] Benatuil L Perez JM Belk J Hsieh CM. An improved yeast transformation method for the generation of very large human antibody libraries. Protein Eng Des Sel. (2010) 23:155–9. doi: 10.1093/protein/gzq002 20130105

[37] Wels W Harwerth IM Zwickl M Hardman N Groner B Hynes NE. Construction, bacterial expression and characterization of a bifunctional single-chain antibody-phosphatase fusion protein targeted to the human erbB-2 receptor. Biotechnol (N Y). (1992) 10:1128–32. doi: 10.1038/nbt1092-1128 1369487

[38] Oberoi P Kamenjarin K Ossa JFV Uherek B Bönig H Wels WS. Directed differentiation of mobilized hematopoietic stem and progenitor cells into functional NK cells with enhanced antitumor activity. Cells. (2020) 9:811. doi: 10.3390/cells9040811 32230942 PMC7226771

[39] Schönfeld K Sahm C Zhang C Naundorf S Brendel C Odendahl M. Selective inhibition of tumor growth by clonal NK cells expressing an ErbB2/HER2-specific chimeric antigen receptor. Mol Ther. (2015) 23:330–8. doi: 10.1038/mt.2014.219 PMC4445620 25373520

[40] Hinz SC Elter A Rammo O Schwämmle A Ali A Zielonka S. A generic procedure for the isolation of pH- and magnesium-responsive chicken scFvs for downstream purification of human antibodies. Front Bioeng Biotechnol. (2020) 8:688. doi: 10.3389/fbioe.2020.00688 32656201 PMC7324474

[41] Oh DY Bang YJ. HER2-targeted therapies - a role beyond breast cancer. Nat Rev Clin Oncol. (2020) 17:33–48. doi: 10.1038/s41571-019-0268-3 31548601

[42] Kwong KY Baskar S Zhang H Mackall CL Rader C. Generation, affinity maturation, and characterization of a human anti-human NKG2D monoclonal antibody with dual antagonistic and agonistic activity. J Mol Biol. (2008) 384:1143–56. doi: 10.1016/j.jmb.2008.09.008 PMC2659651 18809410

[43] Gupta R Brunak S. Prediction of glycosylation across the human proteome and the correlation to protein function. Pac Symp Biocomput. (2002) 7:310–22. 11928486

[44] Howell DN Andreotti PE Dawson JR Cresswell P. Natural killing target antigens as inducers of interferon: studies with an immunoselected, natural killing-resistant human T lymphoblastoid cell line. J Immunol. (1985) 134:971–6. doi: 10.4049/jimmunol.134.2.971 3871222

[45] Kärre K Ljunggren HG Piontek G Kiessling R. Selective rejection of H-2-deficient lymphoma variants suggests alternative immune defence strategy. Nature. (1986) 319:675–8. doi: 10.1038/319675a0 3951539

[46] Pfeifer Serrahima J Zhang C Oberoi P Bodden M Röder J Arndt C. Multivalent adaptor proteins specifically target NK cells carrying a universal chimeric antigen receptor to ErbB2 (HER2)-expressing cancers. Cancer Immunol Immunother. (2023) 72:2905–18. doi: 10.1007/s00262-023-03374-x PMC10412657 36688995

[47] Curio S Jonsson G Marinović S. A summary of current NKG2D-based CAR clinical trials. Immunother Adv. (2021) 1:ltab018. doi: 10.1093/immadv/ltab018 34604863 PMC8480431

[48] Ferrari de Andrade L Tay RE Pan D Luoma AM Ito Y Badrinath S. Antibody-mediated inhibition of MICA and MICB shedding promotes NK cell-driven tumor immunity. Science. (2018) 359:1537–42. doi: 10.1126/science.aao0505 PMC6626532 29599246

[49] Bruhns P Iannascoli B England P Mancardi DA Fernandez N Jorieux S. Specificity and affinity of human Fcgamma receptors and their polymorphic variants for human IgG subclasses. Blood. (2009) 113:3716–25. doi: 10.1182/blood-2008-09-179754 19018092

[50] Radaev S Kattah M Zou Z Colonna M Sun PD. Making sense of the diverse ligand recognition by NKG2D. J Immunol. (2002) 169:6279–85. doi: 10.4049/jimmunol.169.11.6279 12444134

[51] Fan J Shi J Zhang Y Liu J An C Zhu H. NKG2D discriminates diverse ligands through selectively mechano-regulated ligand conformational changes. EMBO J. (2022) 41:e107739. doi: 10.15252/embj.2021107739 34913508 PMC8762575

[52] Mocquot P Mossazadeh Y Lapierre L Pineau F Despas F. The pharmacology of blinatumomab: state of the art on pharmacodynamics, pharmacokinetics, adverse drug reactions and evaluation in clinical trials. J Clin Pharm Ther. (2022) 47:1337–51. doi: 10.1111/jcpt.13741 PMC9796714 35906791

[53] Gjetting T Gad M Fröhlich C Lindsted T Melander MC Bhatia VK. Sym021, a promising anti-PD1 clinical candidate antibody derived from a new chicken antibody discovery platform. MAbs. (2019) 11:666–80. doi: 10.1080/19420862.2019.1596514 PMC6601539 31046547

[54] Li D Li J Chu H Wang Z. A functional antibody cross-reactive to both human and murine cytotoxic T-lymphocyte-associated protein 4 via binding to an N-glycosylation epitope. MAbs. (2020) 12:1725365. doi: 10.1080/19420862.2020.1725365 32054416 PMC7039627

[55] Yang F Zhao L Wei Z Yang Y Liu J Li Y. A cross-species reactive TIGIT-blocking antibody Fc dependently confers potent antitumor effects. J Immunol. (2020) 205:2156–68. doi: 10.4049/jimmunol.1901413 32887749

[56] Harwerth IM Wels W Marte BM Hynes NE. Monoclonal antibodies against the extracellular domain of the erbB-2 receptor function as partial ligand agonists. J Biol Chem. (1992) 267:15160–7. doi: 10.1016/S0021-9258(18)42160-6 1353079

[57] Rohrbach F Weth R Kursar M Sloots A Mittrücker HW Wels WS. Targeted delivery of the ErbB2/HER2 tumor antigen to professional APCs results in effective antitumor immunity. J Immunol. (2005) 174:5481–9. doi: 10.4049/jimmunol.174.9.5481 15843546

[58] Bogen JP Elter A Grzeschik J Hock B Kolmar H. Humanization of chicken-derived antibodies by yeast surface display. Methods Mol Biol. (2022) 2491:335–60. doi: 10.1007/978-1-0716-2285-8_18 35482199

