# Supplementary material for: Bispecific killer cell engagers employing species cross-reactive NKG2D binders redirect human and murine lymphocytes to ErbB2/HER2-positive malignancies
Source: Front Immunol. 2024 Aug 29;15:1457887. doi: 10.3389/fimmu.2024.1457887 (PMC11390497; doi:10.3389/fimmu.2024.1457887)
Supplement: Supplementary file 1 [file DataSheet1.pdf]

Supplementary Material

Bispecific killer cell engagers employing species cross-reactive NKG2D binders redirect human and murine lymphocytes to ErbB2/HER2-positive malignancies

Jordi Pfeifer Serrahima, Katrin Schoenfeld, Ines Kühnel, Julia Harwardt, Arturo Macarrón Palacios, Maren Prüfer, Margareta Kolaric, Pranav Oberoi, Harald Kolmar, Winfried S. Wels

1 Supplementary Table

Supplementary Table 1. Binding of NKAB antibodies to NK and tumor cells

Sample	hNKAR-NK-92	mNKAR-NK-92	MDA-MB-453	MDA-MB-468
**anti-human IgG**	58.5[a)]	84	10.9	10.3
**hNKAB-ErbB2**	559	99.1	4660	18.5
**scNKAB-ErbB2(1)**	972	187	3902	10.9
**scNKAB-ErbB2(2)**	849	290	3985	10.3
**scNKAB-ErbB2(4)**	962	250	4198	10.9
**scNKAB-ErbB2(7)**	1291	385	3933	10.9
**anti-mouse IgG**	10.3	14.5	10.9	10.9
**mNKAB-ErbB2**	10.3	522	3933	10.3

Binding of bispecific scNKAB-ErbB2 molecules and the indicated control proteins at a concentration of 12.5 nM to NKG2D-CAR expressing hNKAR-NK-92 and mNKAR-NK-92 cells, and ErbB2-positive MDA-MB-453 and ErbB2-negative MDA-MB-468 breast carcinoma cells was analyzed by flow cytometry. [a)]Data are indicated as median fluorescence intensity (MFI). Respective histograms are shown in Figure 3C.

2 Supplementary Figures

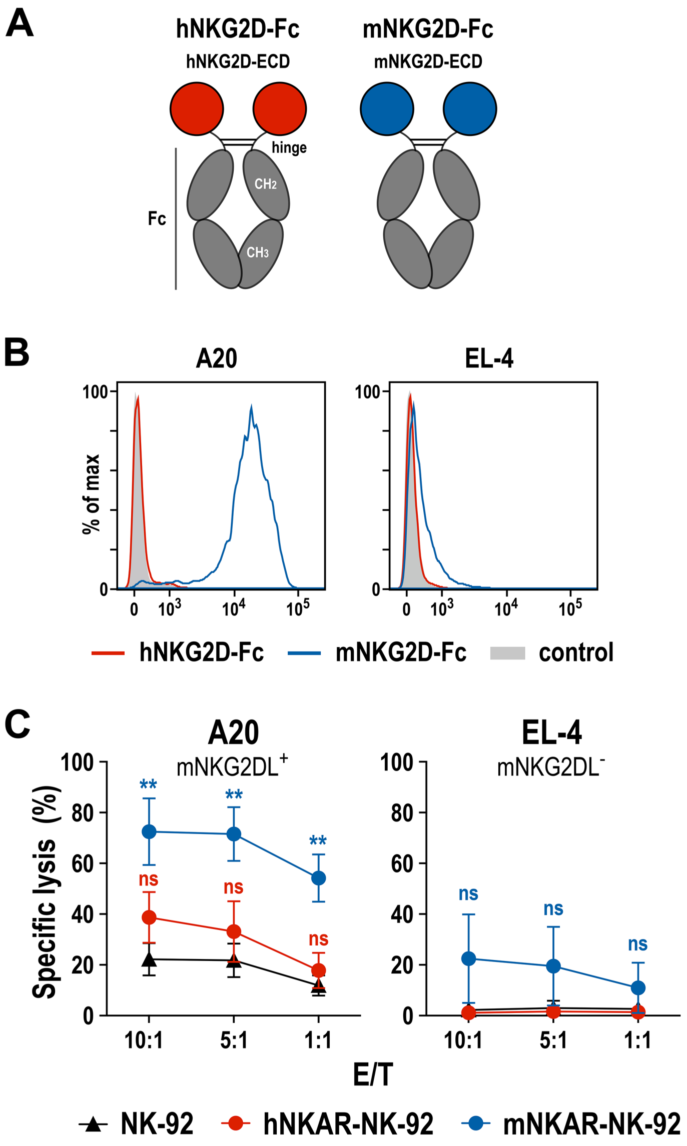

Supplementary Figure 1. Intrinsic activity of mNKAR-NK-92 cells against NKG2DL-positive tumor targets. (**A**) Schematic representation of recombinant hNKG2D-Fc and mNKG2D-Fc proteins, consisting of the extracellular domain of human (red) or murine (blue) NKG2D at the N-terminus, fused to hinge, CH2 and CH3 domains of human IgG4 (Fc). (**B**) Detection of NKG2DL expression on the surface of murine A20 B-cell lymphoma and EL-4 T-cell lymphoma cells by flow cytometry with purified hNKG2D-Fc (red) and mNKG2D-Fc (blue) proteins followed by APC-conjugated anti-human IgG antibody. Cells stained only with secondary antibody (gray areas) served as control. (**C**) Cytolytic activity of mNKAR-NK-92 (blue), hNKAR-NK-92 (red) and parental NK-92 cells (black) against murine A20 and EL-4 cells was determined after 3 hours of co-incubation at the indicated effector to target (E/T) ratios. Mean values ± SD are shown; n=3 independent experiments. **, $p < 0.01$; ns, $p > 0.05$ (not significant). Statistical significance is indicated for differences in comparison to parental NK-92 cells.

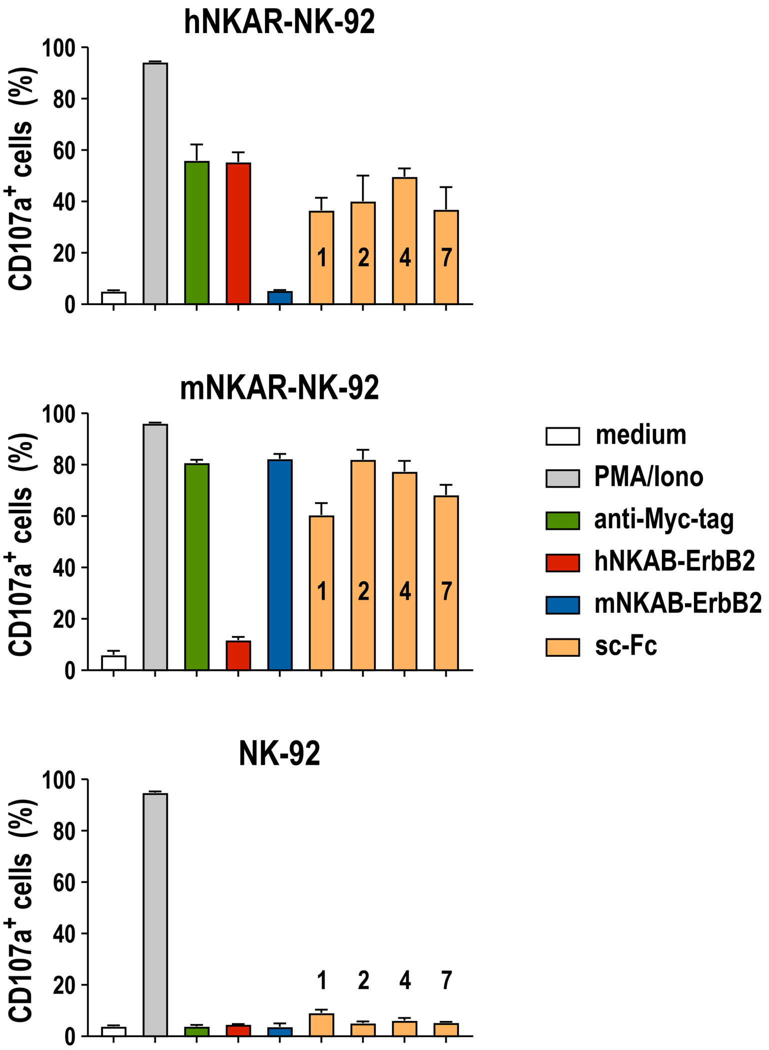

Supplementary Figure 2. Degranulation of NKG2D-CAR expressing NK-92 cells upon activation by species cross-reactive NKG2D binders. hNKAR-NK-92 (top) and mNKAR-NK-92 cells (middle) were stimulated for 4 hours with immobilized scFv-Fc fusion proteins derived from selected yeast display library clones sc1, sc2, sc4 and sc7. Control samples were stimulated with PMA and ionomycin, Myc-tag-specific antibody triggering the NKG2D-CARs in the absence of NKG2D binders, bispecific antibodies solely interacting with human (hNKAB-ErbB2) or murine NKG2D (mNKAB-ErbB2), or were kept in the absence of a stimulator (medium). Parental NK-92 cells were included for comparison (bottom). Mean values ± SD are shown; n=3 independent experiments.

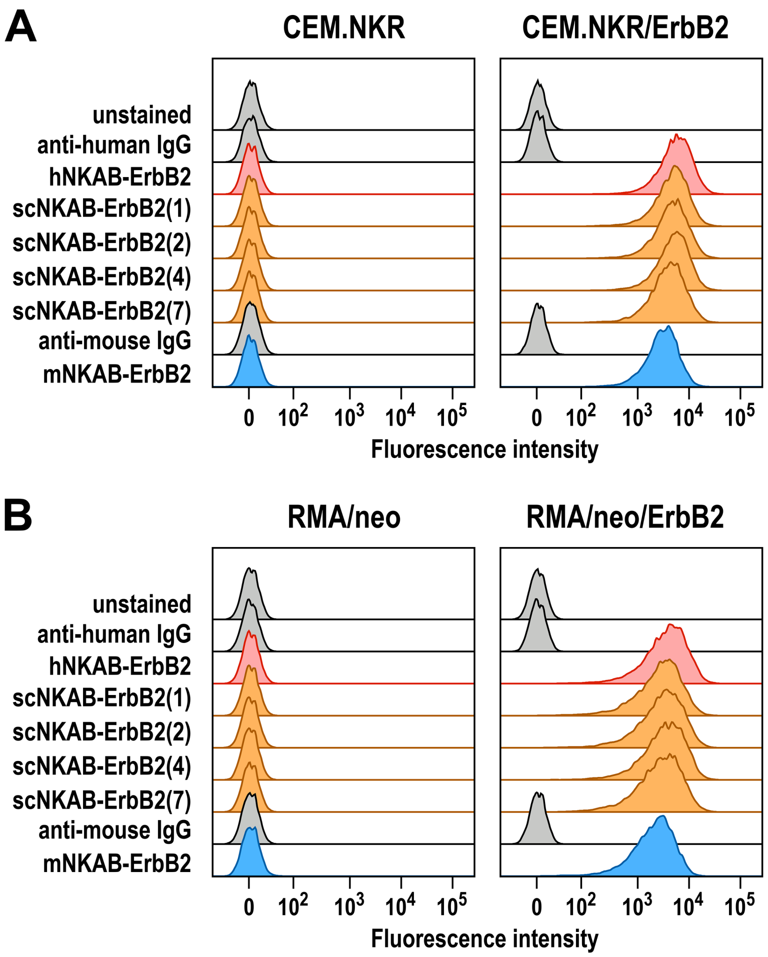

Supplementary Figure 3. Binding of NKAB molecules to ErbB2-expressing human and murine tumor cells. Binding of purified scNKAB-ErbB2, hNKAB-ErbB2 and mNKAB-ErbB2 proteins to human CEM.NKR/ErbB2 (**A**) and murine RMA/neo/ErbB2 T lymphoblastoid cells (**B**) generated by transduction with an ErbB2-encoding lentiviral vector was investigated by flow cytometry as indicated (right panels). Unstained cells and cells only incubated with secondary antibody served as controls. ErbB2-negative parental cells are included for comparison (left panels).

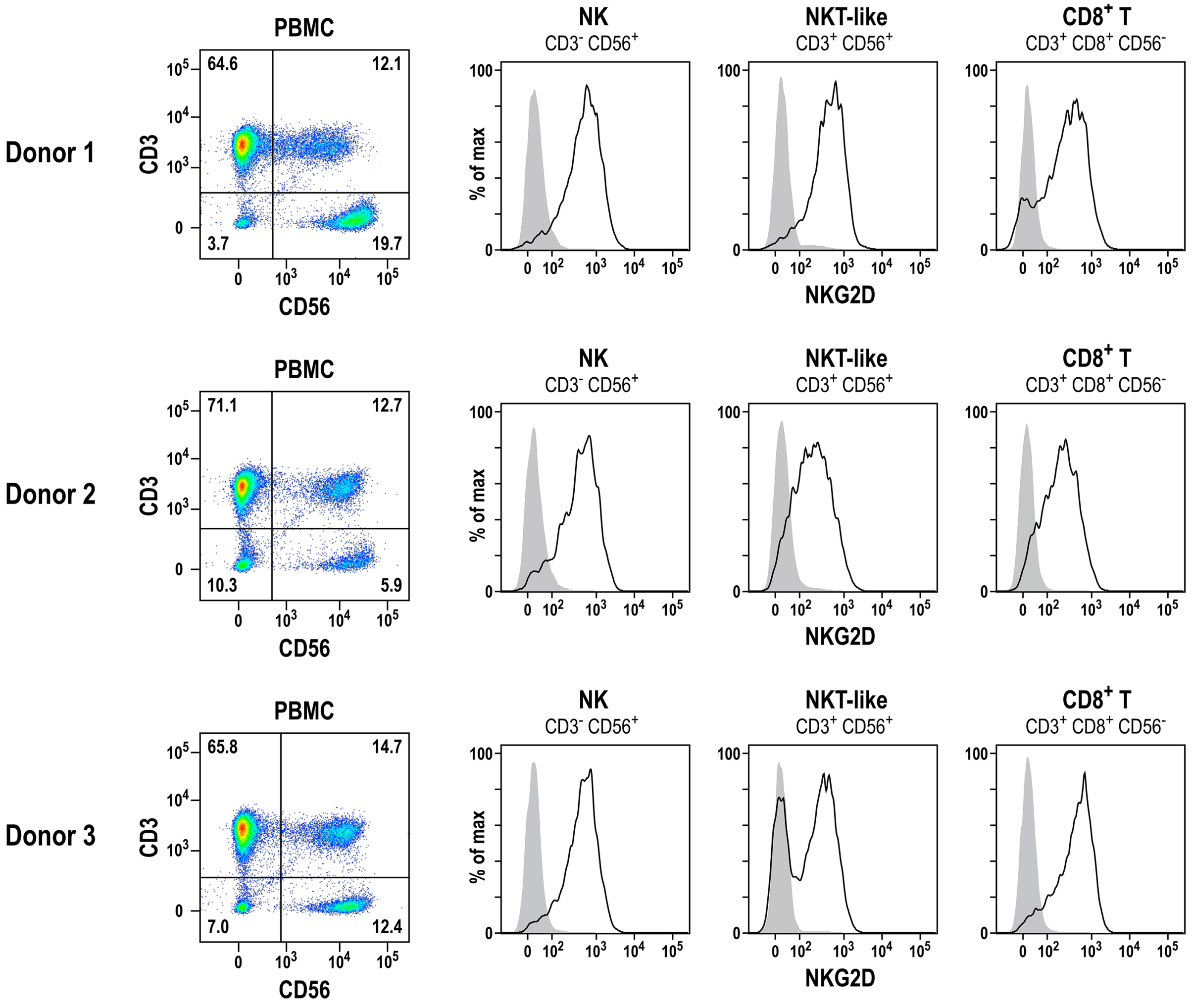

Supplementary Figure 4. Phenotypic characterization of human PBMCs used for the cell killing experiments shown in Figure 7A. PBMCs from three healthy donors were analyzed by multi-color flow cytometry to identify relative proportions of NK ($CD3^{-}$ $CD56^{+}$), NKT-like ($CD3^{+}$ $CD56^{+}$) and T cells ($CD3^{+}$ $CD56^{-}$), and NKG2D surface expression by gated NK, NKT-like and $CD8^{+}$ T cell subpopulations as indicated. Cells stained with the panel antibodies in the absence of anti-NKG2D served as control (gray areas).

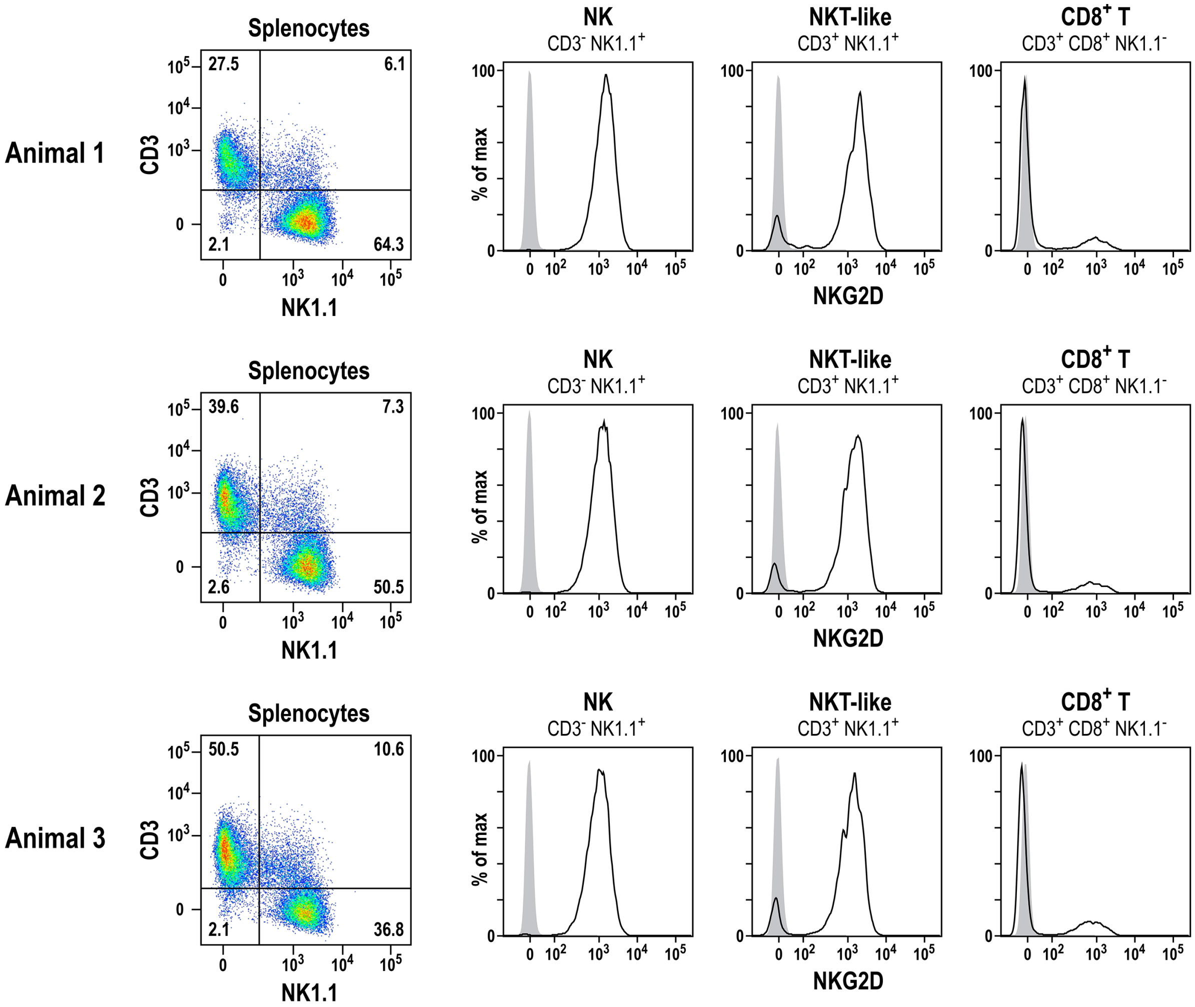

Supplementary Figure 5. Phenotypic characterization of murine splenocytes used for the cell killing experiments shown in Figure 7B. Splenocytes from three individual C57BL/6 mice were analyzed by multi-color flow cytometry to identify relative proportions of NK ($CD3^-$ $NK1.1^+$), NKT-like ($CD3^+$ $NK1.1^+$) and T cells ($CD3^+$ $NK1.1^-$), and NKG2D surface expression by gated NK, NKT-like and $CD8^+$ T cell subpopulations as indicated. Cells stained with the panel antibodies in the absence of anti-NKG2D served as control (gray areas).
